# Large Gas-Phase Source of Esters and Other Accretion Products in the Atmosphere

**DOI:** 10.1021/jacs.2c10398

**Published:** 2023-03-30

**Authors:** Otso Peräkylä, Torsten Berndt, Lauri Franzon, Galib Hasan, Melissa Meder, Rashid R. Valiev, Christopher David Daub, Jonathan G. Varelas, Franz M. Geiger, Regan J. Thomson, Matti Rissanen, Theo Kurtén, Mikael Ehn

**Affiliations:** † Institute for Atmospheric and Earth System Research/Physics, Faculty of Science, University of Helsinki, Helsinki 00014, Finland; ‡ Atmospheric Chemistry Department (ACD), Leibniz Institute for Tropospheric Research (TROPOS), Leipzig 04318, Germany; § Department of Chemistry, University of Helsinki, Helsinki 00014, Finland; ∥ Department of Chemistry, Northwestern University, 2145 Sheridan Road, Evanston, Illinois 60208, United States; ⊥ Aerosol Physics Laboratory, Tampere University, Tampere 33720, Finland

## Abstract

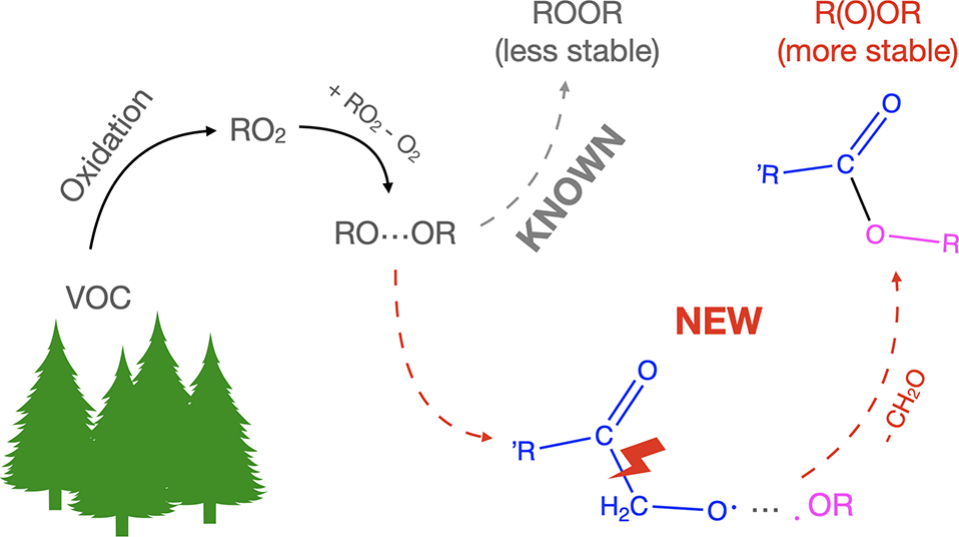

Dimeric accretion products have been observed both in atmospheric aerosol particles and in the gas phase. With their low volatilities, they are key contributors to the formation of new aerosol particles, acting as seeds for more volatile organic vapors to partition onto. Many particle-phase accretion products have been identified as esters. Various gas- and particle-phase formation pathways have been suggested for them, yet evidence remains inconclusive. In contrast, peroxide accretion products have been shown to form via gas-phase peroxy radical (RO_2_) cross reactions. Here, we show that these reactions can also be a major source of esters and other types of accretion products. We studied α-pinene ozonolysis using state-of-the-art chemical ionization mass spectrometry together with different isotopic labeling approaches and quantum chemical calculations, finding strong evidence for fast radical isomerization before accretion. Specifically, this isomerization seems to happen within the intermediate complex of two alkoxy (RO) radicals, which generally determines the branching of all RO_2_-RO_2_ reactions. Accretion products are formed when the radicals in the complex recombine. We found that RO with suitable structures can undergo extremely rapid C–C β scissions before recombination, often resulting in ester products. We also found evidence of this previously overlooked RO_2_–RO_2_ reaction pathway forming alkyl accretion products and speculate that some earlier peroxide identifications may in fact be hemiacetals or ethers. Our findings help answer several outstanding questions on the sources of accretion products in organic aerosol and bridge our knowledge of the gas phase formation and particle phase detection of accretion products. As esters are inherently more stable than peroxides, this also impacts their further reactivity in the aerosol.

## Introduction

Organic peroxy radicals (RO_2_) form in virtually all atmospheric oxidation reactions of volatile organic compounds (VOC) and have key roles in atmospheric chemistry.^1^ Their reactions impact, for instance, radical recycling and the formation of tropospheric ozone and aerosol particles, thus affecting both air quality and climate.^2−5^ Potential reaction pathways of RO_2_ are diverse, ranging from unimolecular isomerisations to bimolecular reactions with several different types of compounds.^6^ The radical structure and environmental conditions govern which pathways will be most favorable.^6^ From the perspective of organic aerosol formation, RO_2_ reaction pathways leading to low-volatile products are of particular interest, as these products can condense onto existing particles or in some cases even form new particles.^7−9^ The primary pathways for achieving such condensable products involve rapidly increasing the oxygen content in the molecules and/or forming accretion products.^4^ As described in more detail below, both pathways have recently been shown to be of greater importance than previously thought.

Rapid incorporation of oxygen can take place through autoxidation, in which an intramolecular hydrogen shift isomerization (H-shift) in the RO_2_ accommodates the addition of molecular oxygen (O_2_).^10^ The resulting RO_2_, now with an additional hydroperoxide functionality, can undergo further isomerization. This can lead to, for example, the formation of closed shell products by ejection of a small radical coproduct such as OH or HO_2_, in a step sometimes referred to as termination.^7,10−12^ Alternatively, the O_2_ addition can be repeated.^7,10−12^ Repeated H-shifts, with the associated O_2_ additions, can lead to the formation of highly oxygenated organic molecules (HOM), which have been closely linked to atmospheric aerosol formation in many systems.^12^

Bimolecular reactions, most commonly with NO, HO_2_, or other RO_2_, are the dominant atmospheric sinks for RO_2_.^6^ Focusing on the RO_2_ self- and cross reactions, current knowledge suggests that a tetroxide intermediate is always formed and rapidly decomposes to a complex of two alkoxy (RO) radicals and a free oxygen molecule:^13−16^
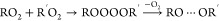
1

The further reactions of the formed alkoxy radical complex determine the resulting products. This complex therefore lies at the core of our study. The main known reaction pathways for this complex involve the direct dissociation of the alkoxy radicals from the complex (eq 2a), an H-shift between the alkoxy radicals followed by dissociation of an alcohol and a carbonyl (eq 2b), or an intersystem crossing (ISC, discussed below) followed by recombination to form an ROOR accretion product (eq 2c):^16^


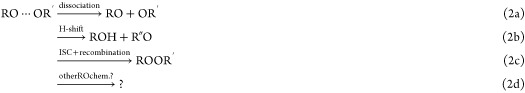
2

In the context of forming low-volatile products, pathways forming accretion products, such as the ROOR (eq 2c), are of particular interest. These types of accretion products, often called dimers,^7^ may have extremely low volatility, and have even been shown to able to form new aerosol particles.^4,9,17−20^ The ROOR pathway was long believed to be a minor one, largely based on studies of small RO_2_.^6^ However, the reaction has since been shown to happen nearly at the collision limit for larger, functionalized RO_2_.^21,22^ As the known products from reaction (2) range from reactive alkoxy radicals to large accretion products, it is already clear that the relative branching ratios are of great importance. The hypothesis of our work was that the lifetime of the RO complex, while short, is still long enough to permit yet other types of RO reactions to take place (eq 2d).

**Scheme 1:**
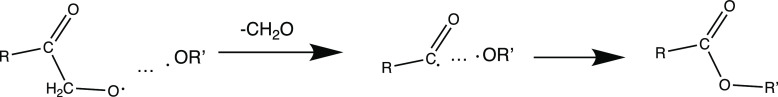
Hypothetical Scission of a β-Oxo-Alkoxy Radical and Subsequent Recombination with Another Alkoxy Radical to Form an Ester

While the experimental evidence for ROOR accretion product formation from RO_2_ self- and cross reactions is very convincing,^7,21,22^ a theoretical description of the process was lacking up until recently. The primary hindrance to the reaction was that the RO···OR′ complex forms with a triplet spin multiplicity, as the O_2_, ejected from the tetroxide, also forms in its triplet ground state.^13−15^ In other words, the two RO radicals will have unpaired electrons with parallel spins, and due to the Pauli exclusion principle, they cannot directly recombine into a singlet ROOR.^15^ However, a spin flip can take place through an intersystem crossing (ISC), at rates often exceeding 10^10^ s^–1^, enabling the ROOR formation.^13,15,23^ This finding is in agreement with experimental evidence on efficient ROOR formation, and overall supports the idea that all RO_2_ self- and cross reactions happen through the RO···OR′ complex.^16^ In extension, if both intermolecular H-shifts (eq 2b) and ISC (eq 2c) are competitive with the dissociation of the RO···OR′ complex, it is possible that also other RO reactions may be fast enough to compete with the dissociation. In particular, β C–C scissions are some of the fastest reported reaction pathways of suitably functionalized RO (ref (24)). If such reactions occur, it may be impossible to experimentally determine whether they occur in the RO while still in complex or in the free RO after dissociation. An exception to this is if the scission occurs in complex, and the products form an accretion product similarly to reaction (eq 2c). In this case the product may no longer contain a peroxide R–O–O–R′ bridge but potentially only a R–O–R′ bridge. Scheme 1 shows such a case, where the first RO radical is expected to undergo a β scission at a rate of 6 × 10^8^ s^–1^, based on a structure–activity relationship (SAR).^24^ If the resulting alkyl radical recombines with the second alkoxy radical, they form an ester. The overall proposed mechanism is analogous to a photochemical process recently characterized in crystalline solids, where a triplet radical pair produced by laser photolysis may undergo CO loss, followed by ISC and recombination.^25^

Accretion products, often esters, have been observed in atmospheric aerosol samples, in particular from reactions of monoterpenes.^26−33^ Various mechanisms, both in the gas- and particle phases, have been suggested for the formation of esters. These include reactions of stabilized Criegee intermediates (sCIs) with carboxylic acids or aldehydes, reactions of RO_2_ with RO, followed by particle phase transformations, and particle-phase Baeyer–Villiger reactions of peroxides with carbonyl species.^32,34,35^ However, no conclusive evidence has been provided for any pathway. As it stands, the ester formation mechanisms remain elusive. Multiple studies have indicated that ozone oxidation of monoterpenes, either by itself or in combination with OH-oxidation, is required for ester formation: they do not seem to form in OH oxidation exclusively.^31,36^ To our knowledge, no gas-phase observations of accretion products identified as esters have been reported.

In this study, we set out to identify if gas phase RO_2_ cross- and self-reactions can lead to other types of accretion products than the previously reported ROOR, for example esters. We hypothesize that the alkoxy radicals in the formed RO···OR′ complex can undergo reactions that still lead to accretion product formation. We studied the ozonolysis of α-pinene, the most abundantly emitted monoterpene,^37^ as monoterpenes are one of the largest sources of organic aerosol globally.^38−41^ We used chemical ionization mass spectrometry (CIMS) as a sensitive method of detecting both RO_2_ radicals and accretion products in the gas phase, with further support gained from different types of isotopic labeling approaches and quantum chemical calculations. These approaches provided good validation for RO_2_ self- and cross reactions being a considerable source of ester accretion products and potentially many other types of accretion products as well.

## Experimental Section

We conducted a series of experiments with both ordinary and isotope-labeled reactants to probe the structures of the accretion products formed during α-pinene ozonolysis. Experiments were performed both at the Leibniz Institute for Tropospheric Research (TROPOS) in Leipzig, Germany and at the University of Helsinki, Finland. In addition to these experiments, we conducted quantum chemical calculations to confirm the plausibility of the proposed mechanisms. Short descriptions of each method are given below, while more detailed descriptions can be found in the Supporting Information (SI).

### Instrumentation

To monitor both radicals and closed shell species, we used a chemical ionization atmospheric pressure interface time-of-flight (CI-APi-TOF) mass spectrometer.^42^ With detection limits on the order of 10^4^ cm^–3^, it is a very sensitive type of CIMS.^42^ In this study, we used the CI-APi-TOF with two different reagent ions: nitrate (NO_3_^–^) and ethylaminium (C_2_H_5_NH_3_^+^). Nitrate is the most commonly used reagent in the CI-APi-TOF, known to be selective toward highly oxygenated species.^43^ Aminium ionization scheme, here, ethylaminium, is much less selective but sensitive for a wide range of oxygenated products and requires clean operating conditions.^43,44^

### Laboratory Experiments

The laboratory experiments were conducted using a free-jet flow tube in TROPOS and a continuous stirred-tank reactor (CSTR, made out of Teflon) “COALA” in the University of Helsinki.

In both systems, ozone and α-pinene were injected to, and products sampled from, the reaction vessel in a continuous manner. The total flow in TROPOS was 100 L min^–1^, making the reaction time 7.9 s, and it was 52 L min ^–1^ in the CSTR, making the residence time around 40 min. During the experiments in the COALA chamber, we varied both the ozone and α-pinene concentrations and thus the oxidation rate. With the long residence times of the chamber, the oxidation rate clearly affects the product distribution.

At low oxidation rates, RO_2_ radicals make up a larger fraction of the total measured signal.^7,45^ When moving to higher oxidation rates, this fraction decreases, as closed shell products become more dominant.^7,45^ For our results, we used both high and low oxidation rates: low for clear RO_2_ signals, unperturbed by neighboring closed shell products, and high oxidation rates for higher signal-to-noise ratio for the accretion products. During the measurements, we had issues with drifting sensitivity of the CI-APi-TOF; therefore, exact signal intensities are not directly comparable across experiments.

### Isotopic Labeling

Mass spectrometry readily provides the elemental composition of a molecule. In order to obtain additional structural information on the molecules of interest, we conducted three types of isotope experiments.

**Figure 1:**
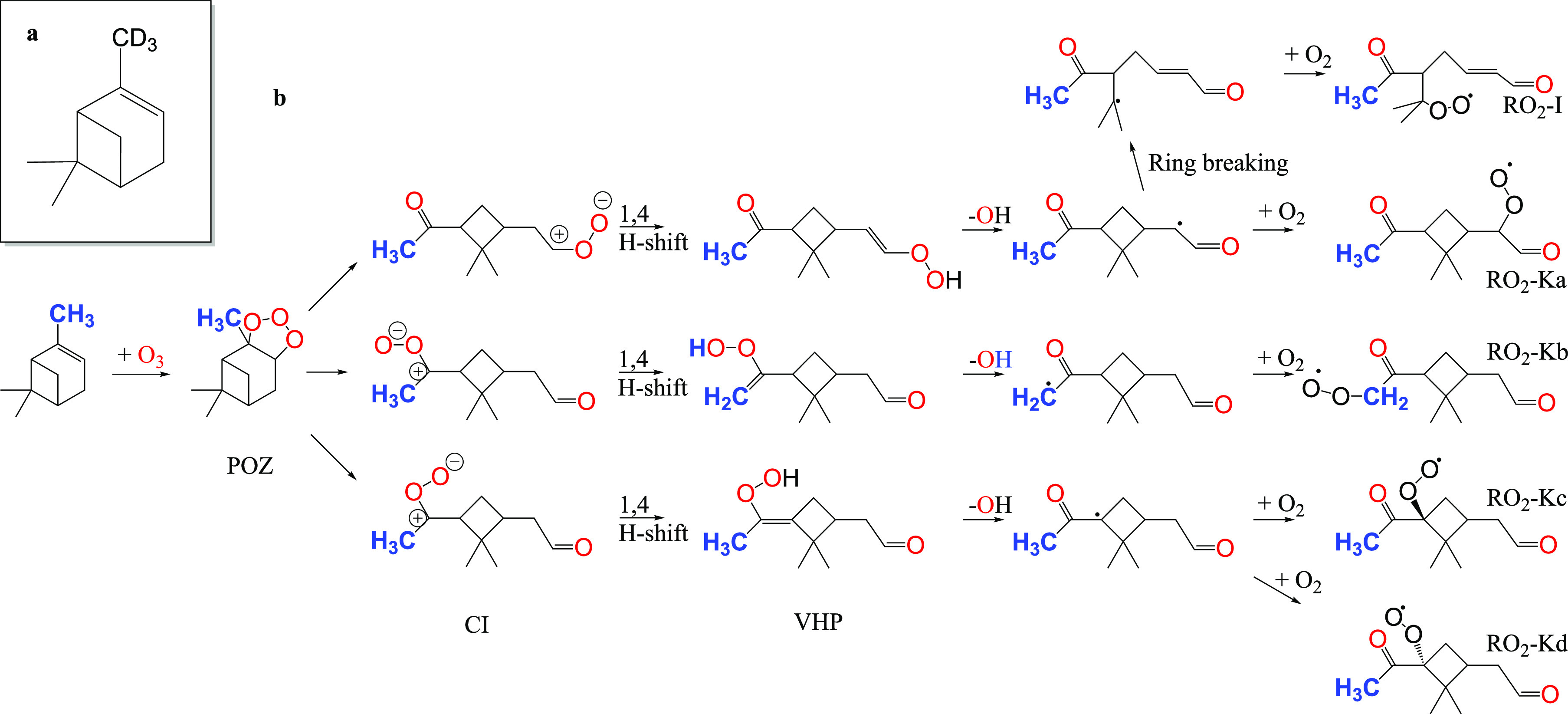
(a) Structure of the selectively deuterated α-pinene. (b) Nonexhaustive reaction mechanism, showing the formation of the five known^48,49^ C_10_H_15_O_4_ RO_2_ radicals in the ozonolysis of α-pinene. In addition to the shown products, many other types are also formed. The naming of the RO_2_ radicals is the same as in Kurtén et al.,^49^ with the addition of the RO_2_–I, which corresponds to the RB1-RO_2_ from Iyer et al.^48^ The methyl group that is deuterated in (a) is colored blue in the reactions. The oxygen atoms incorporated from the ozone addition are colored red. Labeled are also the primary ozonide (POZ), Criegee intermediates (CIs), and vinyl hydroperoxides (VHP).

1. First, we added heavy water (D_2_O) to the COALA chamber during certain experiments. In the presence of excess D_2_O, any exchangeable hydrogen atoms, i.e., H atoms bound to O atoms, were switched to deuterium. This is observed as a shift by one mass unit for every exchanged H in the detected ions. This has been used previously to determine the number of hydroperoxide functional groups in HOM.^46,47^ Those studies revealed that typical HOM from α-pinene ozonolysis most likely contain a peroxide bridge,^47^ as the number of exchangeable H atoms was one fewer than expected from autoxidation. This assertion is supported by later theoretical work.^48^ We also performed experiments with ordinary water (H_2_O) to verify that the addition of water vapor did not interfere with the chemistry in other ways (see the SI for details).
2. In the second method, we re-examined earlier TROPOS α-pinene ozonolysis experiments,^7^ where the ozone was in the form of ^18^O_3_, with a focus on specific accretion products. For each O atom in the molecule deriving from ^18^O_3_, a shift of two mass units will be observed compared to a similar elemental composition with only ^16^O atoms. Ozonolysis proceeds via several rapid steps, starting with the formation of a primary ozonide (POZ) and subsequently a Criegee intermediate (CI).^6^ The CI can then react through the so-called vinylhydroperoxide (VHP) channel, where it forms a VHP after an intramolecular hydrogen shift and proceeds to eject an OH radical. This is followed by an O_2_ addition to form an RO_2_ (Figure 1b, ref (6)). As a result, the first peroxy radicals, of the formula C_10_H_15_O_4_, contain two O atoms from ozone and two from an added oxygen molecule. At least five distinct RO_2_ radicals of the formula C_10_H_15_O_4_ are known to form in the oxidation of α-pinene by ozone (Figure 1).^48,49^
3. In the third type of experiments, we used a form of α-pinene with a deuterated methyl group (Figure 1 a) at the COALA chamber. The synthesis of the deuterated α-pinene isotopologue was accomplished using the procedure previously reported by Upshur et al.^50^ In this case, the deuterated precursor is 3 Da heavier than usual. Similarly, any oxidation products retaining all the deuterium will be 3 Da heavier than their normal counterparts. If the deuterated C atom is lost during the oxidation, the three deuterium atoms attached to it are lost as well, which will be visible in the mass spectrum. In addition, if a deuterium atom is abstracted during autoxidation, it becomes bound to an O atom in a hydroperoxide group, thus becoming labile.^47^ In our experiments, enough water vapor was present in the system to exchange all the labile deuteriums to hydrogens (−OOD + H_2_O → – OOH + HDO; see the SI for details). Thus, each exchange of D to H in a compound, with a corresponding mass shift by 1 Da, means of a C–D bond was broken during the oxidation process.

### Quantum Chemistry

We conducted quantum chemical calculations to assess whether our proposed reaction mechanism is feasible. We sampled the conformers of relevant alkoxy and alkyl radicals using the Spartan 18 program.^51^ The complexes of these radicals were systematically sampled building up on the approach described by Kubečka et al.^52^ Conformers were sampled also for the product ROR accretion products. We calculated the ISC rated for both ^3^(RO···OR′) and ^3^(R″···OR′) complexes. For a more detailed description of the computational methodology, see the SI.

In addition to these calculations, the rate of the RO_2_–Kb alkoxy bond scission reaction was determined computationally to refine the estimate from the SAR. Conformational sampling was implemented in a similar fashion as outlined above. The reaction rate was calculated using the Eyring equation; for more details, see SI.

## Results and Discussion

Ozonolysis of α-pinene leads to the formation of RO_2_ radicals with the formula C_10_H_15_O_4_ (Figure 1). In the process, an OH radical is released; thus, ozonolysis is typically accompanied by OH oxidation, mainly forming RO_2_ radicals of the formula C_10_H_17_O_3_ (Figure S1).^6,53−55^ Potential autoxidation can increase the O atom content in steps of two for each radical, but both C atom and H atom content, as well as O atom parity (i.e., whether the radical has an even or odd number of oxygen atoms), will remain unchanged. Consequently, the expected ROOR accretion products from this system will have the compositions C_20_H_30_O_even_, C_20_H_32_O_odd_, or C_20_H_34_O_even_, depending on the combination of the RO_2_ from the different oxidants (see also Table S1). In the most comprehensive mapping of products from this system to date, Berndt et al. detected all these radicals and accretion products, in addition to other expected closed-shell monomeric species.^22^ However, the most abundant accretion products were in fact C_19_H_28_O_odd_ and C_19_H_30_O_even_. The authors concluded that these could be nominally explained by the loss of CH_2_O from the expected accretion products, yet no mechanistic explanation was provided. Berndt et al. did observe a nine-carbon RO_2_ radical, C_9_H_15_O_5_, as well.^22^ In addition to the measured concentrations being extremely low, this RO_2_ has only lost CO, not CH_2_O. From this follows that it should not be responsible for the formation of the highly abundant C_19_H_28_O_odd_ and C_19_H_30_O_even_ accretion products. These accretion products behaved kinetically like the C_20_ accretion products, with a square dependency on the reaction rate. This indicates that their formation only requires one RO_2_–RO_2_ reaction. Therefore, it is plausible that they would form through RO_2_ self- and cross-reactions with a CH_2_O loss occurring in the RO···OR′ complex, analogous to Scheme 1.

### Accretion Product Formation in the TROPOS Flow Reactor

**Scheme 2:**
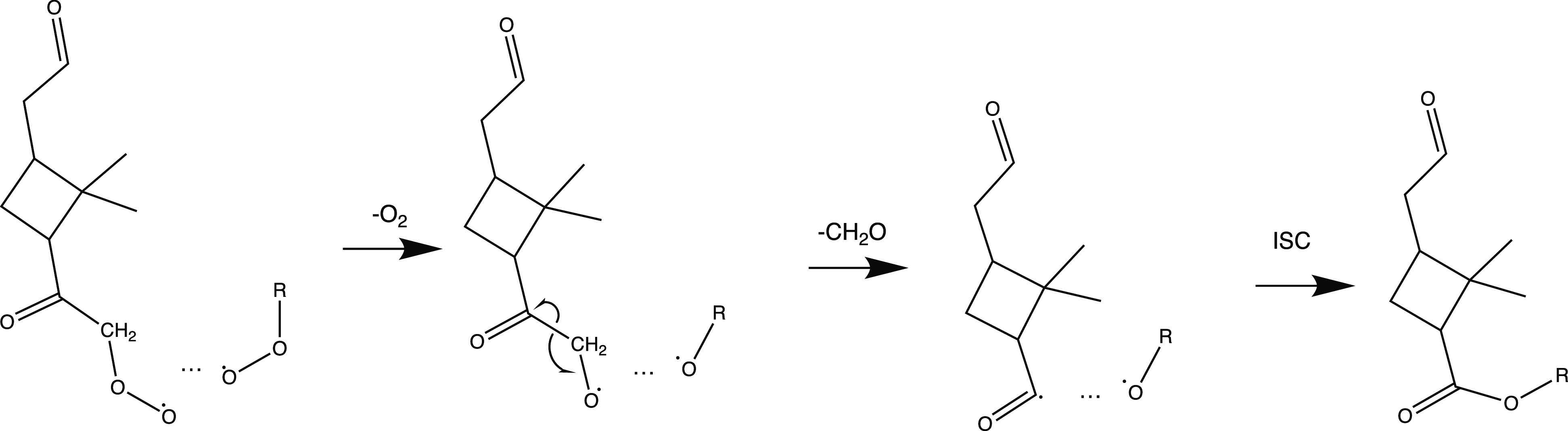
Tentative Reaction Schematic of the RO_2_–RO_2_ Reaction Leading to the Formation of C_19_ Accretion Products, Including the Loss of CH_2_O and Recombination of the Resulting Alkyl Radical Together with the RO Radical The reacting RO_2_ with the structure shown is RO_2_–Kb from Figure 1.

A first key result from Berndt et al.^22^ is the lack of C_19_H_32_O_odd_ species, which would be expected to form from two OH-derived RO_2_, accompanied by CH_2_O loss upon accretion. Experiments with only OH oxidation confirm that these types of C_19_ accretion products do not form in pure OH oxidation.^22^ Taken together, these findings indicate that only O_3_-derived RO_2_ are susceptible to this type of loss. The primary RO_2_ radicals formed in ozonolysis have the formula C_10_H_15_O_4_ (Figure 1 b), and the expected accretion products (eq 2c) from their cross- and self-reactions are C_20_H_30_O_6_ or following a loss of CH_2_O, C_19_H_28_O_5_. Both of these were observed at high concentrations by Berndt et al.^22^ The RO_2_–RO_2_ reaction between two C_10_H_15_O_4_ forms a complex of RO radicals (eq 2a). For the RO formed from each of the five C_10_H_15_O_4_ isomers (Figure 1b), in only one would a β scission lead to the loss of CH_2_O. This is the RO_2_–Kb, with a structure analogous to the one in Scheme 1. The fates of the other RO_2_ are discussed later. For the scission to take place in the RO···OR complex, it would need to be competitive with the other possible reaction pathways, meaning an order of magnitude of 10^6^ s^–1^ or more.^16^ Utilizing a structure–activity relationship (SAR),^24^ we can estimate a rate for this scission reaction to be 6 × 10^8^ s^–1^, which is high enough to be potentially competitive. As such, we can proceed with the hypothesis that a large fraction of the C_19_ accretion products are formed after loss of formaldehyde from RO_2_–Kb. The resulting acyl radical would then reside in an R″···OR′ complex, still in a triplet state since the formaldehyde will form in a singlet state. If these radicals are able to recombine, through ISC or otherwise, they can form an ester accretion product. This mechanism of formaldehyde loss and subsequent ester formation is summarized in Scheme 2.

The above process, together with O_2_ additions due to autoxidation, can explain all the accretion products observed by Berndt et al.,^22^ save for two (C_20_H_30_O_4_ and C_20_H_30_O_5_) that were detected at low intensities. It is also worth noting that the four most abundant accretion products observed by Berndt et al. (C_19_H_28_O_5_, C_19_H_30_O_8_, C_19_H_28_O_9_, and C_19_H_28_O_7_) have all undergone loss of CH_2_O. On one hand, this means this type of loss occurs frequently, and thus, its mechanisms are of importance. On the other hand, it also means that either the yield of RO_2_–Kb is much greater than the 41% (out of all C_10_H_15_O_4_ RO_2_ radicals), suggested by Master Chemical Mechanism (MCM^56,57^), RO_2_–Kb forms accretion products more efficiently than other RO_2_ or that many other radicals are also able to undergo similar scission reactions. Further validation is therefore needed, first to prove the underlying hypothesis and second to test whether other types of CH_2_O loss are expected. Our isotope experiments are optimal for this purpose.

### Isotope-Labeled Reactants

**Figure 2:**
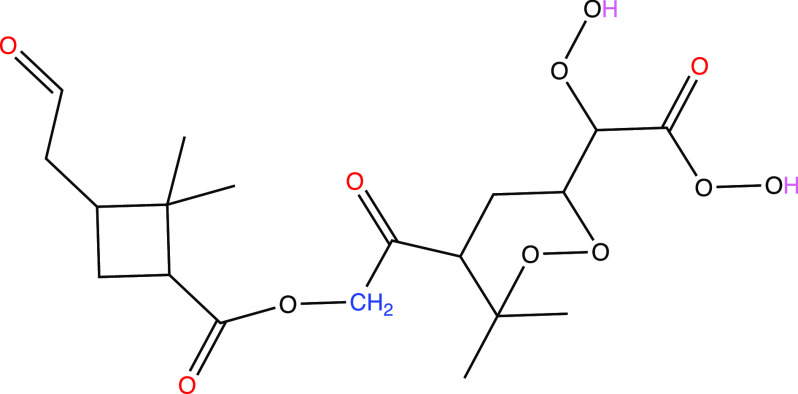
Hypothetical structure of a C_19_H_28_O_11_ ester. We have assumed that C_19_H_28_O_11_ forms in the reaction of RO_2_–Kb with C_10_H_15_O_10_ according to Scheme 2. C_10_H_15_O_10_ was assumed to have a structure corresponding to the C_10_H_15_O_8_ suggested by Iyer et al.,^48^ with an additional H-shift and O_2_ addition. The O atoms added from the initial ozone attack are colored red, while the labile H atoms expected to undergo exchange with gaseous water vapor (H_2_O or D_2_O) are colored pink. The C atom, along with the attached hydrogens, in the deuterated methyl group (Figure 1 a) is colored blue. RO_2_–Kb is assumed to have lost this C atom, and therefore, only one such C atom is left in the molecule. Note that the exact structure of the right side subunit of the accretion product is only an illustrative example, and we do not expect the actual structure to exactly match it.

In this section, we will concentrate on identifying key details about the C_19_ accretion product structures using isotope-labeled reactants. Due to instrumental limitations, all experiments here were conducted using nitrate ionization, meaning that we can only observe the accretion products that have undergone autoxidation. We posit that the systematic steps of O_2_ in the data by Berndt et al. suggest that there is a good correspondence between the peaks in each individual series, e.g., C_19_H_28_O_5,7,9,11_. Remarkably, the highest oxygen numbers for the C_19_ accretion products are lower than for the C_20_ counterparts; as a result, the vast majority of them could be formed in the reactions of RO_2_–Kb with C_10_H_15_O_4,6,8,10_ RO_2_ radicals (SI for details). The focus lies here on the C_19_H_28_O_11_ accretion product, as that is readily detected with nitrate ionization, and typically the highest accretion product peak in α-pinene ozonolysis nitrate CIMS spectra.^7,47,58^ Note that other, less oxygenated accretion products are even more abundant but not detected with nitrate ionization.^22^ To make the upcoming interpretations easier to follow, we have drawn a hypothetical structure for C_19_H_28_O_11_, formed in the reaction of RO_2_–Kb together with a C_10_H_15_O_10_ RO_2_ radical (Figure 2). The structure of the C_10_H_15_O_10_ radical can only be guessed, and probably our structure is not an exact match. However, it does match observations reported earlier, which show that it should only have two labile H atoms, suggesting that an endoperoxide bond is included in the molecule.^47^

**Figure 3:**
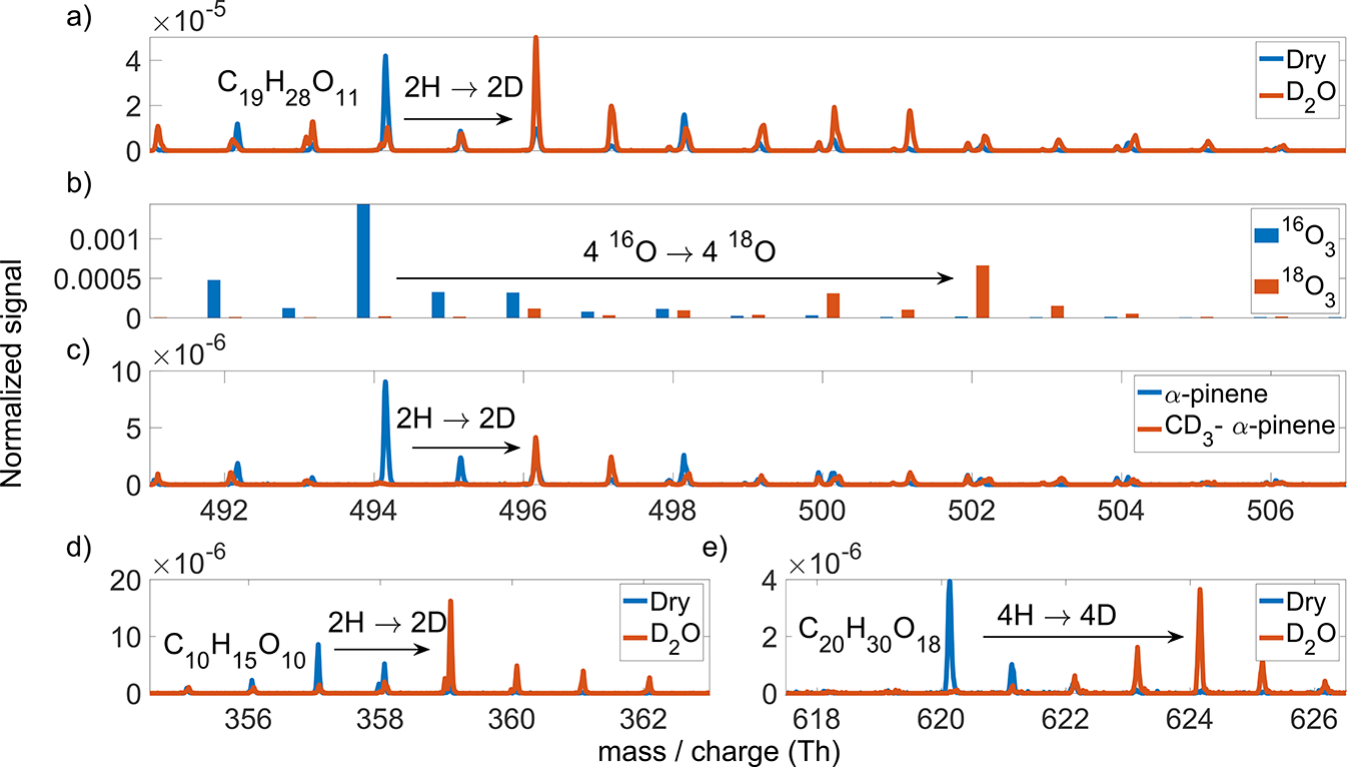
Comparison of experiments with isotope-labeled reactants (red) with ordinary reactants (blue) during α-pinene ozonolysis. (a) Shift of two Th upon humidification with D_2_O indicates that C_19_H_28_O_11_ has two exchangeable hydrogen atoms. (b) When oxidized by heavy ozone (^18^O_3_), C_19_H_28_O_11_ shifts by eight mass units as compared to oxidation with ordinary ozone (^16^O_3_). This signifies that all four of the oxygen atoms (two per α-pinene derived subunit, as one per subunit is lost in an OH radical) from the initial ozone addition remain in the accretion product. (c) Shift of two mass units in the experiments with the selectively deuterated α-pinene indicates that C_19_H_28_O_11_ retains only two of the initial six deuterium atoms. (d) C_10_H_15_O_10_ shifts by two mass units upon humidification by D_2_O, indicating two exchangeable hydrogen atoms. (e) C_20_H_30_O_18_, formed in the reaction of two C_10_H_15_O_10_, has four exchangeable hydrogens, meaning that the number is conserved upon accretion. All data were measured with nitrate CI-APi-TOF, with a, c, d, and e in the COALA chamber and b at TROPOS, using an older flow tube.^7^ All peaks are observed clustered with the nitrate ion. Reactant concentrations: (a), [α-pinene] = 20 ppb, [O_3_] = 90 ppb; (b), [α-pinene] = 80 ppb, [O_3_] = 15 ppb; (c), [α-pinene] = 5 ppb, [O_3_] = 90 ppb; (d), [α-pinene] = 3 ppb, [O_3_] = 15 ppb; (e), [α-pinene] = 20 ppb, [O_3_] = 90 ppb. (b) is in unit mass resolution, with the bars offset for clarity. Other subplots in high resolution. Normalized signal: the signal divided by the sum of the reagent ion (NO_3_^–^, (HNO_3_)NO_3_^–^, (HNO_3_)_2_NO_3_^–^) signals.

During α-pinene ozonolysis experiments in the COALA chamber, the addition of D_2_O caused C_19_H_28_O_11_ to shift by two mass units (Figure 3a), i.e., conversion into C_19_H_26_D_2_O_11_. This suggests two exchangeable hydrogen atoms, in accordance with the structure in Figure 2. This also agrees with the observed shift by two mass units for the observed C_10_H_15_O_10_ radical (Figure 3d), implying that the other RO_2_ (in this case RO_2_–Kb) would not have any labile H atoms. Similarly, the accretion product C_20_H_30_O_18_, expected to form in the self-reactions of C_10_H_15_O_10_, shifts by the expected four mass units (Figure 3e), indicating that the labile H atom count does not change during the accretion.

Re-examining our previous ^18^O_3_ experiments,^7^ we confirmed that C_19_H_28_O_11_ contains four oxygen atoms from the initial ozone additions (Figure 3b), which is in agreement with our hypothesized structure (four carbonyl groups in Figure 2). Specifically, this finding also means that the O atom lost in CH_2_O has to be from an O_2_ that has added to the molecule after the ozone attack.

Finally, to confirm the carbon atom lost in the C_19_ accretion products, we conducted experiments with the selectively deuterated α-pinene shown in Figure 1a. The labeled C atom is the one that has the peroxy moiety in RO_2_–Kb (Figure 1b) and would thus be lost in a reaction similar to the one in Scheme 2. Therefore, we would expect a considerable loss of D in the C_19_ accretion products. We found that autoxidation alone lead to the loss of a maximum of one deuterium atom per precursor α-pinene molecule (SI for details). In C_19_H_28_O_11_, there were only two deuterium atoms left (Figure 3c), meaning that four have been lost from the two initial C_10_H_13_D_3_ precursors. Taken together, these two findings indicate that one of the subunits has lost one deuterium in autoxidation and subsequent exchange with H_2_O, while the other subunit has lost the entire carbon atom to which the deuterium atoms were bound. Put together, we conclude that our hypothesized ester linkage (Figure 2) has very strong support and that the most abundant accretion products in the α-pinene ozonolysis system are, in fact, esters formed through the cross-reactions of RO_2_ radicals.

### Further Validation and Potential Other Accretion Product Types

As a final validation of this mechanism (Scheme 2), we conducted quantum chemical calculations to confirm the plausibility of the different steps in the mechanism.

For the reaction to happen as hypothesized, multiple conditions must be fulfilled:

1. The β scission of one RO in the RO···OR′ complex must be fast enough to compete with the other reaction pathways in Reaction (2), namely,
  (a) the dissociation of the complex
  (b) the intermolecular H-shift to form an alcohol and a carbonyl
  (c) or the recombination through ISC to ROOR.
2. The resulting R″···OR′ complex must also have a long enough lifetime to enable the recombination to R″OR′.
3. To recombine as singlet, an ISC must happen fast enough to compete with the dissociation of the radical complex.
4. Finally, for the recombination to happen, it has to be thermodynamically favorable.

To verify these steps are possible, we computed the β scission rates of the RO radical formed from RO_2_–Kb (Condition 1), bond strengths of the RO···OR′ complexes to verify they are long-lived enough for one to fragment (Condition 1), the spin flip rates in said complexes to verify fragmentation can compete with the spin flip (Condition 1), ^3^(R···OR′) stabilities (Condition 2), ^3^(R···OR′) spin flip rates (Condition 3), and thermodynamics of ^1^(ROR) and ^3^(ROR) formation (Condition 4).

Quantum chemical calculations based on ωB97X-D/aug-cc-pVTZ-level energies (SI for details) give a scission rate of 2 × 10^9^ s^–1^ for the RO radical formed from RO_2_–Kb, even higher than the 6 × 10^8^ s^–1^ estimated from SAR. This strengthens the assertion that the RO_2_–Kb-derived RO radical can undergo a β scission while in the RO···OR′ complex. Calculating the dissociation rates for the RO···OR′ and R″···OR′ complexes is not straightforward (see the SI). However, the dissociation is associated with a relatively high (>10 kcal/mol in energy) binding energy. As a result, the RO scission rate is likely to be competitive with the dissociation of the complex (again, see the SI for details). Published intermolecular H-shift rates are typically on the order of 1 × 10^9^ s^–1^ or below.^16^ As a result, the H-shift is probably not a dominant pathway, also evidenced by the abundant observations of accretion products. We calculated the ISC rate for the ^3^(RO···OR′) complex to be up to 2 × 10^8^ s^–1^. Again based on the abundant formation of accretion products,^22^ ISC is assumed to be competitive with the dissociation of the ^3^(RO···OR′) complex. Thus, the scission (with a rate of 2 × 10^9^ s^–1^, as compared to the ISC rate of up to 2 × 10^8^), should also be competitive. In total, based on the calculated and published reaction rates for competing reactions, the β scission seems to be competitive enough to occur often, satisfying Condition 1.

We calculated the ISC rate for the ^3^(R″···OR′) complex to be up to 7 × 10^11^ s^–1^. This is very fast, meaning that, if such a complex forms, ISC to the singlet state (and the subsequent recombination) is very likely a competitive pathway. Unsurprisingly, recombination on the singlet surface is extremely exergonic (Δ*G* = −83 kcal/mol, SI for details). Taken together, these computational results support our mechanism for the formation of the ^1^(R″OR′) accretion product. Interestingly, the recombination to R″OR′ would be energetically favorable even on the triplet surface, due to the adjacent carbonyl bond being able to accommodate an excited state without breaking (SI for details). However, test calculations at the ωB97X-D/aug-cc-pVTZ level on a four-carbon model system (triplet CH_3_–C·(O)····O–CH_2_–CHO, obtained by taking two C atoms from either side of the C–O–C bond in Figure 2 and replacing groups further away by H atoms) indicate that this process likely has an energy barrier of over 10 kcal/mol. This barrier effectively prevents the reaction, and accretion likely happens through the fast ISC.

**Figure 4:**
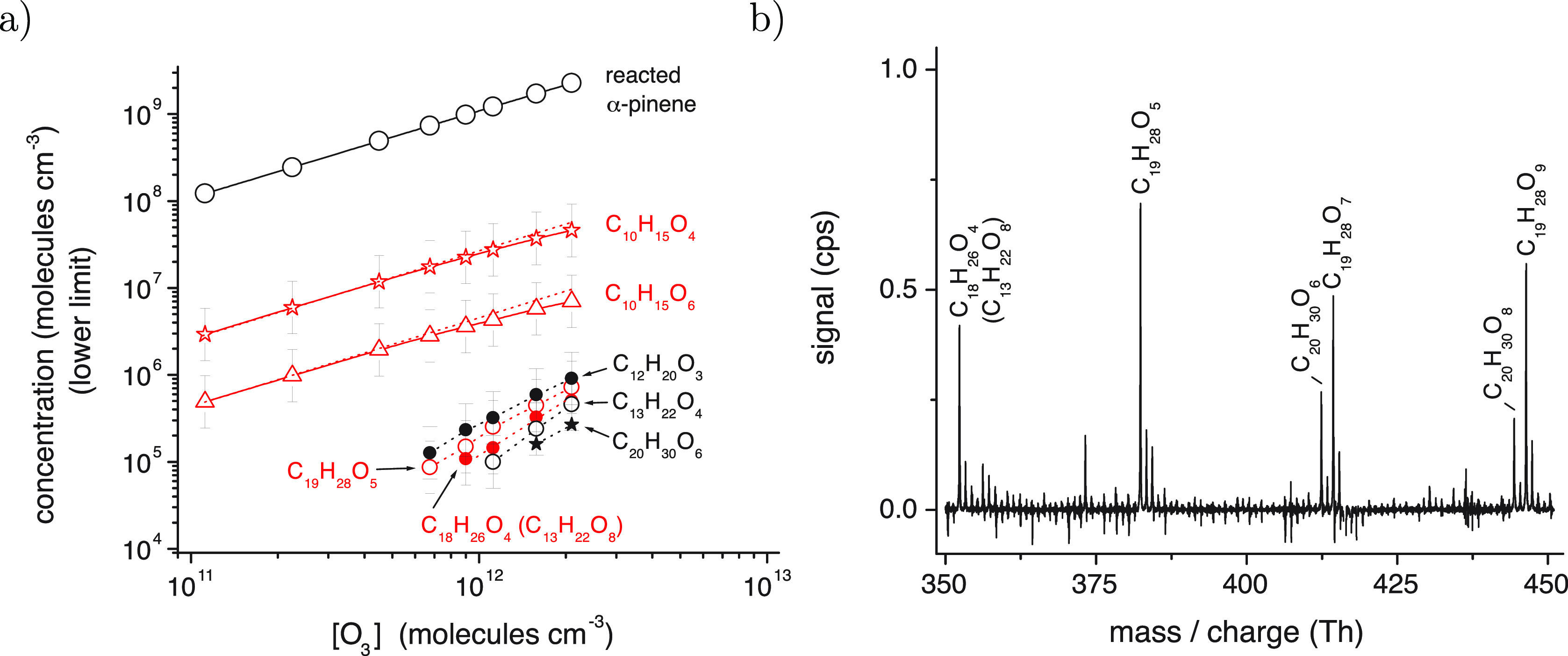
(a) Formation of selected RO_2_ radicals, C_10_H_15_O_4_ and C_10_H_15_O_6_, and accretion products from the ozonolysis of α-pinene in the presence of propane for OH scavenging. Product sampling was carried out by means of a dilution unit (dilution gas: high purity nitrogen) with a dilution factor of 7, not considered in the stated concentrations. Reactant concentrations were [α-pinene] = 1.25 × 10^12^, [propane] = 1.23 × 10^16^, and [O_3_] = (1.12–21) × 10^11^ molecules cm^–3^. (b) Product spectrum (background corrected) from the ozonolysis of α-pinene as measured for the highest α-pinene conversion of the experiment depicted in panel a. The products appear as adducts with C_2_H_5_NH_3_^+^, i.e., with a signal shift by 46.09 Th in the spectrum. All data measured in TROPOS.

With our experimental and computational results strongly supporting our suggested mechanism of ester formation, we can attempt additional prediction. If the C_19_ products form through fragmentation of RO_2_–Kb (C_10_H_15_O_4_, Figure 1 b) in the RO···OR′ reaction complex, we would expect ester formation in the reactions of RO_2_–Kb with RO_2_ radicals from other precursor VOCs as well. In some experiments at TROPOS, propane was added during α-pinene ozonolysis to act as an OH scavenger. The reaction of propane with OH yields an RO_2_ with the formula C_3_H_7_O_2_. It has been observed already earlier that C_3_H_7_O_2_ reacts with C_10_H_15_O_4_ to form C_13_H_22_O_4_ accretion products.^22^ However, the accretion product C_12_H_20_O_3_, expected to form following CH_2_O elimination, is indeed observed and is even more abundant than C_13_H_22_O_4_ (Figure 4 a), again indicating an important role of this new mechanism. Further, analogous C_14_ and C_11_ products are observed in mixtures of α-pinene with both isoprene and ethylene, respectively, as well (see the SI), as expected.

The abundant observations of the C_12_ and C_19_ accretion products suggests that the β scissions are highly competitive with all other reaction pathways. As such, one might even expect that in some reactions between two RO_2_–Kb, both undergo scissions in the RO···OR′ complex. If followed by recombination, the resulting product would be C_18_H_26_O_4_. While overlapping with another ion (see the SI), this product was also observed with the same kinetic behavior as the other accretion products (Figure 4). The C_18_ product was not reported earlier, as even at moderate concentrations, it is affected by instrumental artifacts. Therefore, the experiment shown in Figure 4 was performed utilizing a dilution unit before the CIMS to avoid these.^59^ The artifacts are presumably due to RO_2_ reactivity being enhanced by reagent ions in the chemical ionization inlet, in a similar fashion to what has been observed for sulfuric acid ion cluster formation.^60^ MD simulations based on a recent model for RO_2_ + R′O_2_ overall rates also supports the hypothesis that clustering with ions substantially increases the rate for the least oxidized RO_2_^61,62^ (see SI for more details). With this limitation in mind, and the dilution system in use, we observed even the C_18_H_26_O_4_ accretion product to form more abundantly than the nonscissioned C_20_H_30_O_6_ accretion product, supporting the assertion that such scissions are common and important.

Interestingly, this C_18_ accretion product would then form from the recombination of two alkyl radicals, effectively forming an alkyl accretion product, i.e., two subunits attached through a C–C bond. Thus, the mechanism of in-complex RO scissions followed by recombination is likely not limited to forming esters. In the case of α-pinene oxidation by OH, the resulting RO_2_ are hydroxy peroxy radicals (Figure S1). If these form alkoxy radicals, the bond between the alkoxy and hydroxy C atoms is expected to scission extremely fast, forming a alkyl radical with the radical center on the hydroxy C atom (see the SI for more details). If recombining with an RO, this would lead to a hemiacetal accretion product. Similarly, RO_2_-Kc and RO_2_-Kd have fast scission reactions available when forming the corresponding RO radicals, where the four-membered ring would break to form a tertiary alkyl radical (SI). Reaction with a (nonacyl) RO would in this case produce an ether accretion product. Importantly, the scission reactions forming ethers or hemiacetals discussed here would only break a ring structure and thus not lead to fragmentation. As a result, the formed accretion products would retain all of the carbon atoms and have the same elemental composition as the ROOR accretion products. Therefore, we have no easy way to test this hypothesis experimentally, like in the case where ester formation was accompanied by formaldehyde loss. Nevertheless, we deem these structures feasible, meaning that they could make up a fraction of the accretion products thus far attributed to peroxides. Final (in)validation of this will require further studies.

### Atmospheric Implications

Ester accretion products are inherently less reactive than peroxide accretion products.^63,64^ Therefore, the direct gas phase formation pathway may be of particular importance if the stability of these accretion products in the condensed phase is drastically higher. A large fraction of studies that have identified accretion product structures in organic aerosol from monoterpenes have identified esters as the dominant accretion product type.^28−31^ The formation of esters has been speculated to take place through different mechanisms, either in particle phase or in gas phase.^32,34,35^ Even if formed in the gas phase, it is possible that further reactions take place in the particles as the gas-phase oxidation products, such as the majority of those described in this work, often contain reactive hydroperoxide functionalities.^12^ The reported particle-phase accretion products often contain shorter carbon chains than observed in our study.^31,36^ Nevertheless, Kristensen et al. did detect several C_19_H_28_O_*n*_ accretion products during α-pinene ozonolysis experiments,^33^ which may have been formed through the reactions presented in this work, possibly followed by particle phase conversion of reactive hydroperoxide groups to less reactive forms. This would be in line with the observations that the particle phase accretion products do not contain hydroperoxide groups.^65^

Ester accretion products have been observed to require ozone oxidation to form.^31,36^ It has also been found that, while OH oxidation alone does not produce ester accretion products, the formation of some of the ester accretion products is suppressed when introducing an OH scavenger.^31,36^ These features have been explained by suggesting that stabilized Criegee intermediates (sCIs) would be required for ester formation.^31^ This mechanism has also received criticism, for example, due to the fact that esters are observed also at high relative humidities, when sCIs predominantly react with water vapor.^36^ Our proposed mechanism explains how ozone oxidation is required and how OH oxidation can also affect ester formation. Only the ozone-derived RO_2_ will have a suitable structure to form ester accretion products after the in-complex scission of the resulting RO radical. Analogous scission reactions that may be expected in OH-initiated RO_2_ would lead to hemiacetal formation. As such, the observed ozone dependence of ester formation can be explained without sCIs involvement. Some ester accretion products, such as the abundant C_19_H_30_O_8_, can form in the reaction between RO_2_–Kb with an RO_2_ from OH oxidation. As a consequence, their formation is inhibited upon OH scavenger addition.^22^

While we have only studied α-pinene ozonolysis in this study, the suitable structure for β scission to form an acyl radical, namely, a peroxy group and a carbonyl on adjacent C atoms, is ubiquitous in alkene ozonolysis for all but the smallest alkenes. We therefore expect that this route to gas-phase esters should be important for a large fraction of biogenic VOCs. As was seen with the mixtures of α-pinene with propane, isoprene, and ethylene, the other reacting RO_2_ does not need to be large or highly functionalized or originate from α-pinene oxidation. The role of other partner RO_2_ radicals, such as the abundant CH_3_O_2_, in similar reactions remains an open question.

One remaining open question concerns the fact that Berndt et al. did not observe any large signals of closed-shell C_9_ products.^22^ We would expect these to form from the RO undergoing CH_2_O loss, whether in the alkoxy complex or after dissociation, and ultimately terminating in some other way than accretion. One possibility is that the scission and recombination are always so fast that ester formation has a yield of nearly 100%, i.e., never allowing the dissociation of the RO in this case. The dissociation energy for the alkoxy complex is over 2-fold higher than that of the alkoxy bond scission barrier energy (14 vs 5 kcal/mol; SI for details), indicating that the dissociation pathway may be too slow to be important. However, the same scission-prone RO radical should also form in the reaction of RO_2_ with NO, but no large C_9_ signals were observed in this system either. For now, this remains the main inconclusive result for which we do not have an unambiguous explanation.

## Conclusion

We performed in-depth experiments to deduce the kinetics and structural features of accretion products formed from RO_2_ cross- and self-reactions. With the premise that an intermediate RO···OR′ complex forms in this reaction,^15,16^ our aim was to deduce to which extent the alkoxy radicals can undergo intramolecular reactions before recombining into an accretion product. We studied α-pinene ozonolysis, utilizing highly sensitive chemical ionization mass spectrometers in our laboratory studies. We combined several methods of isotopic separation as well as quantum chemical calculations to identify and validate the relevant reaction mechanisms.

As our main finding, β C–C bond scission of one of the most abundant primary RO_2_ from α-pinene ozonolysis was highly competitive in the RO···OR′ complex. As a result, the majority of accretion products observed in this system formed after recombination of an acyl radical and an alkoxy radical, producing ester accretion products. Thus, in addition to the well-known peroxide accretion products, gas-phase RO_2_ self- and cross-reactions can also be a large source of ester accretion products. We can further speculate that also several other types of accretion products may form following reactions within the radical complex, including ether, hemiacetal, and alkyl accretion products. For the latter, we saw indications of β scissions in both RO before recombining, in which case the C_18_ product would simply be connected through a C–C bond. We identified pathways also to the other accretion product types, but as they do not include fragmentation, we cannot verify this experimentally with our methods.

Exactly which types of accretion products will form, if any, will ultimately depend on the exact structures of the reacting RO_2_ radicals, and we expect that this mechanism of “in-complex” RO reactions may be relevant, yet overlooked, in many different systems. In particular, the ozonolysis of alkenes, which comprise most biogenic organic emissions, should have a large yield of ester accretion products under conditions where RO_2_ self- and cross-reactions are important. As esters are known to be more stable than peroxides, this new pathway is of particular importance for forming long-lived low-volatile accretion products for atmospheric aerosol formation.

## Data Availability

Full mass spectra of the data showin in Figure 3 and quantum chemical calculations can be found at https://zenodo.org/record/7701889#.ZCGeCXbMKUk.

## Supporting Information Available

Discussions of initial RO_2_ radical formation in OH oxidation, accretion product formation patterns, laboratory experiments, quantum chemical calculations, interpretation of the D shift spectra, nonideal behaviour of certain accretion products in dilution experiments, and scissions of other RO, figures of formation of the three initial RO_2_ radicals in OH oxidation of α-pinene, spectra of accretion products, state averaged CASSF (10,10)/6-311++G(d,p) molecular orbitals, optimized structures, HOM spectra, product spectrum of the α-pinene ozonolysis, minimum energy configurations, and scission products for the RO radicals, and tables of compositions of C_20_ accretion products, obtained accretion products and their signal intensities, main signals of accretion products and their intensities, relative electronic energies and Gibbs free energies, ISC rate constants, association time of complexes between RO_2_ species and predicted reaction rates, and scission rates for the RO scission reactions (PDF)
