# Supplementary material for: Large Gas-Phase Source of Esters and Other Accretion Products in the Atmosphere
Source: J Am Chem Soc. 2023 Mar 30;145(14):7780–90. doi: 10.1021/jacs.2c10398 (PMC10103131; doi:10.1021/jacs.2c10398)
Supplement: Supplementary file 1 — ja2c10398_si_001.pdf [file ja2c10398_si_001.pdf]

Supplementary information for "A large gas-phase source of esters and other accretion products in the atmosphere"

Otso Peräkylä,*,† Torsten Berndt,‡ Lauri Franzon,¶,† Galib Hasan,¶,† Melissa Meder,† Rashid R. Valiev,¶,† Christopher David Daub,¶,† Jonathan G. Varelas,§ Franz M. Geiger,§ Regan J. Thomson,§ Matti Rissanen,¶,† Theo Kurtén,¶,† and Mikael Ehn*,†

†*Institute for Atmospheric and Earth System Research / Physics, Faculty of Science, University of Helsinki, Helsinki 00014, Finland*

‡*Atmospheric Chemistry Department (ACD), Leibniz Institute for Tropospheric Research (TROPOS), 04318 Leipzig, Germany.*

¶*Department of Chemistry, University of Helsinki, Helsinki 00014, Finland*

§*Department of Chemistry, Northwestern University, 2145 Sheridan Road, Evanston, Illinois 60208, United States*

E-mail: otso.perakyla@helsinki.fi; mikael.ehn@helsinki.fi

Contents

1 Initial RO_2 radical formation in OH oxidation **3**

2 Accretion product formation patterns **3**

3 Laboratory experiments **5**

3.1 Accretion products detected at the TROPOS flow tube 6

3.2 Examples of accretion product spectra 7

4 Quantum chemical calculations 16

4.1 Methods 16

4.1.1 Conformational Sampling of Isolated alkoxy and alkyl radicals 16

4.1.2 Systematic sampling of the $^3(RO \cdots OR)$ and $^3(R \cdots OR)$ clusters . . 17

4.1.3 Sampling of the ROR ester accretion product 18

4.1.4 ISC rate Calculation 19

4.1.5 Alkoxy Bond Scission Rate Calculation 20

4.2 Results and discussion 21

4.2.1 ISC rate calculation 23

4.2.2 Stability of the ROR ester accretion product 24

4.2.3 Alkoxy Bond Scission Rate Calculation 24

5 Interpretation of the D shift spectra: labile H, max number of abstracted D from CD3 AP. 26

5.1 Exchange of labile deuteriums to hydrogen with H_2O 26

5.2 Maximum number of deuterium lost in the autoxidation of d3-α-pinene . . . 28

6 Non-ideal behaviour of certain accretion products in dilution experiments 28

6.1 Molecular Dynamics Simulations of Interactions Between α-Pinene Derived Peroxy Radicals and the Ethyl-ammonium Cation $CH_3CH_2NH_3^+$ 31

7 Scissions of other RO 33

References 34

1 Initial RO_2 radical formation in OH oxidation

RO_2 radicals are formed in the OH oxidation of α-pinene as well: as compared to ozonolysis, the reaction proceeds more straightforwardly[1]. A simplified schematic on the formation of the three initial RO_2 radicals is shown in Fig. S1.

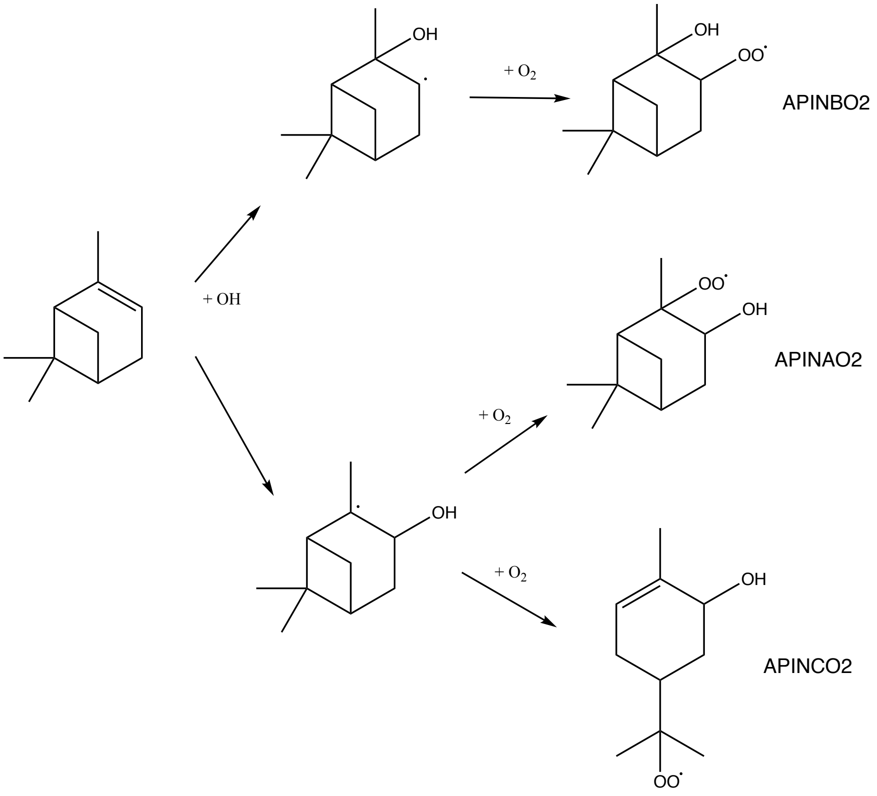

Figure S1: Formation of the three initial RO_2 radicals in OH oxidation of α-pinene, adapted from Xu et al. and Rolletter et al..[2,3] Again, the mechanism is not exhaustive, but only to show the formation of the known RO_2 radicals with the formula $C_{10}H_{17}O_3$.

2 Accretion product formation patterns

The first organic peroxy radicals formed in α-pinene ozonolysis have the formula $C_{10}H_{15}O_4$. Some of these can undergo autoxidation, in which O_2 molecules are sequentially added to the peroxy radical to yield a more highly oxygenated peroxy radical with the formula $C_{10}H_{15}O_{4+2n}$. Here n denotes the number of autoxidation steps. Correspondingly, in the OH

oxidation of α-pinene, the first peroxy radicals have the formula $C_{10}H_{17}O_3$, and $C_{10}H_{17}O_{3+2m}$ upon autoxidation. Peroxy radicals can react together to form ROOR accretion products (and an O_2 molecule). The different possible combinations of the peroxy radicals generated in α-pinene ozonolysis and OH reactions to yield a ROOR accretion product are listed in Table S1.

Table S1: Compositions of C_{20} accretion products formed from RO_2 radicals generated in the O_3 or OH initiated autoxidation of α-pinene ($C_{10}H_{16}$). The n,m, p and q denote the number of autoxidation steps each radical has gone through: with no autoxidation, these are equal to zero. All of the precursor RO_2 and the resulting accretion products, have been observed[4]. The highest oxygenated compound for each type of RO_2 and accretion product observed were $C_{10}H_{15}O_{10}$, $C_{10}H_{17}O_7$, $C_{20}H_{30}O_{18}$, $C_{20}H_{32}O_{15}$ and $C_{20}H_{34}O_{12}$. The highest oxygen numbers show a clear correspondence between the RO_2 and the accretion products: e.g. two of the highest oxygenated RO_2 from ozonolysis,$C_{10}H_{15}O_{10}$, are expected to form $C_{20}H_{30}O_{18}$, which was the highest oxygenated accretion product observed. The numbers correspond to n and p up to 3 and m and q up to 2.

	$\mathbf{C_{10}H_{15}O_{4+2n}}$ ($C_{10}H_{16}$ + O_3)	$\mathbf{C_{10}H_{17}O_{3+2m}}$ ($C_{10}H_{16}$ + OH)
$\mathbf{C_{10}H_{15}O_{4+2p}}$ ($C_{10}H_{16}$ + O_3)	$\mathbf{C_{20}H_{30}O_{6+2(n+p)}}$	$\mathbf{C_{20}H_{32}O_{5+2(m+p)}}$
$\mathbf{C_{10}H_{17}O_{3+2q}}$ ($C_{10}H_{16}$ + OH)	$\mathbf{C_{20}H_{32}O_{5+2(n+q)}}$	$\mathbf{C_{20}H_{34}O_{4+2(m+q)}}$

In addition to C_{20} accretion products, Berndt et al. also observed $C_{19}H_{28}O_{odd}$ and $C_{19}H_{30}O_{even}$ accretion products. The molecular formulae correspond to the C_{20} accretion products on the first row of Table S1, with the elimination of an additional CH_2O. As compared to the C_{20} accretion products, the C_{19} accretion products had lower oxygen numbers. The majority of them, and all of the most abundant ones, can be explained with the reactions of $C_{10}H_{15}O_4$ with either $C_{10}H_{15}O_{4+2n}$ (to yield $C_{19}H_{28}O_{odd}$) or $C_{10}H_{17}O_{3+2m}$ (to yield $C_{19}H_{30}O_{even}$) (Table S2).

Table S2: Similar to Table S1, but including an elimination of CH_2O, and with $C_{10}H_{15}O_4$ always being the other reaction partner.

	$\mathbf{C_{10}H_{15}O_{4+2n}}$ ($C_{10}H_{16}$ + O_3)	$\mathbf{C_{10}H_{17}O_{3+2m}}$ ($C_{10}H_{16}$ + OH)
$\mathbf{C_{10}H_{15}O_4}$ ($C_{10}H_{16}$ + O_3)	$\mathbf{C_{19}H_{28}O_{5+2n}}$	$\mathbf{C_{19}H_{30}O_{4+2m}}$

3 Laboratory experiments

The experimental investigations at TROPOS have been conducted in a free-jet flow system at T = 295 ± 2 K, a pressure of 1 bar with a total flow of 100 l min^{-1} (STP) of purified air and a reaction time of 7.9 s. More detailed information on the experimental setup is given elsewhere.[5,6] Ozone was produced by passing 2 l min^{-1} (STP) air through an ozone generator (UVP OG-2). α-pinene was taken from a gas mixture in helium prepared in a gas-metering unit. All gas flows were set by means of calibrated gas flow controllers (MKS 1259/1179). Ozone concentrations were measured at the outflow of the reactor by a gas monitor (Thermo Environmental Instruments 49C) and α-pinene by means of a PTR mass spectrometer (HS PTR-QMS 500, Ionicon).[7]

Detection of reaction products was conducted using a CI-APi-TOF mass spectrometer (Airmodus, Tofwerk) sampling from the centre flow of the free-jet flow system with a rate of 10 l min^{-1} (STP). The ion-molecule reaction (IMR) proceeded at atmospheric pressure using a Boulder-type inlet system.[8] Product ionisation was carried out by means of $C_2H_5NH_3^+$ (ethylaminium).[6] Stated lower limit concentrations were obtained using a calibration factor $f = 1.85 \times 10^9$ molecules cm^{-3}. The "lower limit" approach assumes collision limit in the ion-molecule reaction and no ion losses within the mass spectrometer.[6]

The experiments at the University of Helsinki were carried out in the COALA chamber, a two cubic meter (2 m^3) continuous stirred-tank reactor (CSTR) made out of teflon (FEP) foil. The setup is described in more detail elsewhere,[9,10] but a brief overview is given here. Purified air, along with reactants, is continuously injected into the chamber at a total rate of 51.5 l min^{-1}. Nearly the same amount of air is continuously sampled, with the excess amount flushed out of the chamber to maintain a slight overpressure in the chamber. We injected the isotope-labelled α-pinene in liquid phase into a small (0.5 l min^{-1}) flow of high purity nitrogen using a syringe pump with variable injection speeds. The α-pinene evaporated into the flow, and entered the chamber in the gas phase. We produced ozone using an ozone generator, and humidified the flow of clean air into the chamber in select experiments by

bubbling it through either purified (MilliQ) water or heavy water (D_2O).

3.1 Accretion products detected at the TROPOS flow tube

If the RO radical originating from RO_2-Kb undergoes a scission in the complex of two alkyl radicals to yield the C_{19} ester accretion products, we would expect this scission to occur also when RO_2-Kb is in complex with various other RO_2 radicals. We found this to be true at least for RO_2 generated from the oxidation of isoprene and ethylene as well: in these cases, the non-scissioned accretion products contain 15 and 13 carbon atoms, respectively. However, after the loss of CH_2O in the beta scission, we expect accretion products with 14 and 12 carbon atoms: both were observed, as expected (Figs. S2 and S3). These accretion products were overlooked earlier in the study by Berndt et al.[4].

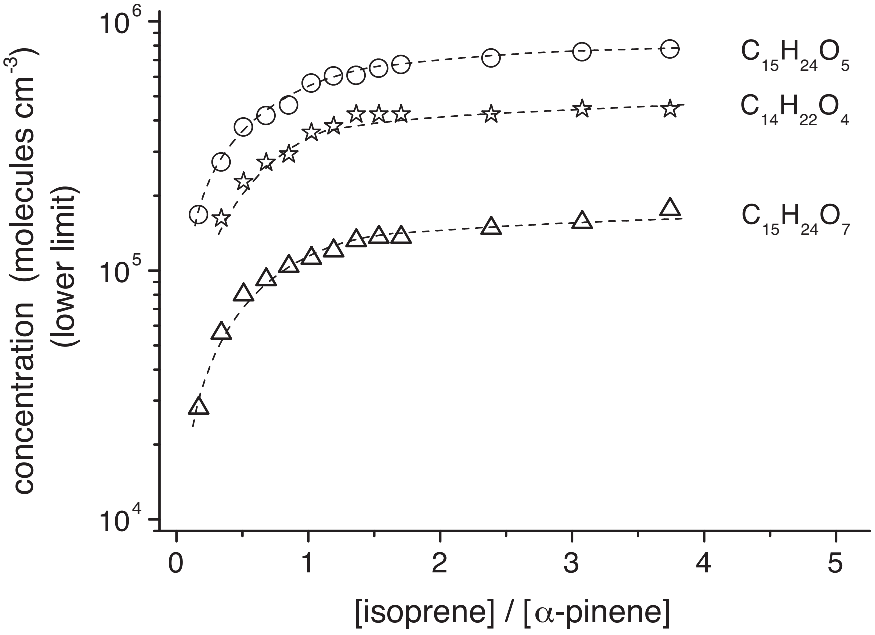

Figure S2: Formation of accretion products $C_{14}H_{22}O_4$, $C_{15}H_{24}O_5$ and $C_{15}H_{24}O_7$ from the reaction of $HO-C_5H_8O_2$ radicals with ozonolysis-derived C_{10} radicals as a function of the isoprene/α-pinene ratio (reanalysis of data given in Berndt et al..[4] The measurements have been conducted by means of $C_3H_7NH_3^+$ ionization. Reactant concentrations were $[O_3] = 2.0 \times 10^{12}$, $[\alpha\text{-pinene}] = 3.9 \times 10^{11}$ and $[\text{isoprene}] = (0 - 1.46) \times 10^{12}$ molecules cm^{-3}.

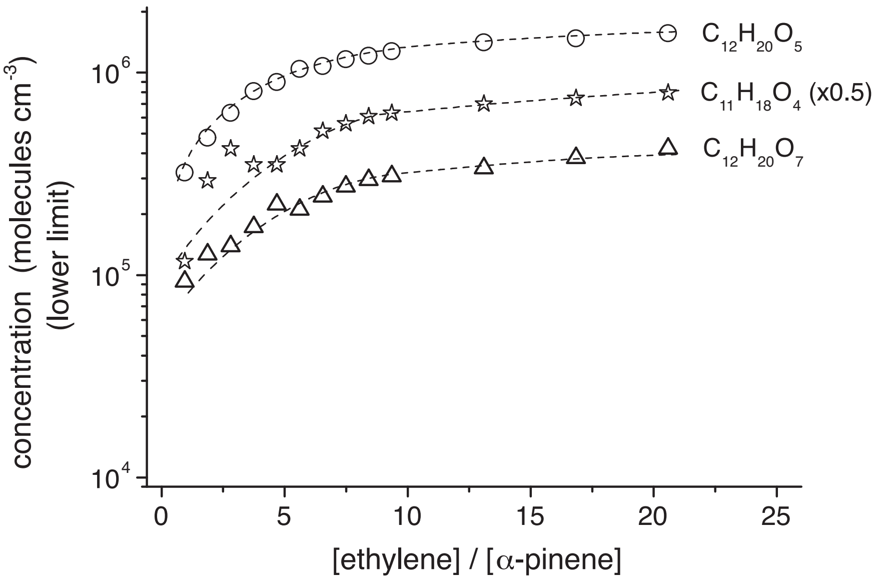

Figure S3: Formation of accretion products $C_{11}H_{18}O_4$, $C_{12}H_{20}O_5$ and $C_{12}H_{20}O_7$ from the reaction of HO-C2H4O2 radicals with ozonolysis-derived C10 radicals as a function of the ethylene/α-pinene ratio (reanalysis of data given in Berndt et al..[4] The measurements have been conducted by means of $C_3H_7NH_3^+$ ionization. Reactant concentrations were $[O_3] = 2.0 \times 10^{12}$, $[\alpha\text{-pinene}] = 3.9 \times 10^{11}$ and [ethylene] = (0 - 8.03) $\times 10^{12}$ molecules cm^{-3}.

3.2 Examples of accretion product spectra

We have included tables and spectra of accretion products detected both in in the TROPOS flow tube and the COALA chamber (Tables S3 – S7 and Figs. S4 – S7). Tentative formation mechanisms, including the reactant RO_2 radicals and the assumed linkage type, for the most abundant accretion products are summarized in Table S8.

Table S3: Obtained accretion products ($C_2H_5NH_3^+$ adducts) and their signal intensities from the ozonolyis of α-pinene measured in the free-jet flow system at TROPOS applying product dilution by a factor of 7, see spectrum in Fig. 4b of the main manuscript. Reactant concentrations were: [α-pinene] = 1.25×10^{12} molecules cm^{-3}, [propane] = 1.23×10^{16} molecules cm^{-3} and [O_3] = 2.1×10^{12} molecules cm^{-3}.

Composition	Nominal adduct mass (product)$C_2H_5NH_3^+$ (Th)	Normalized signal *
$C_{20}H_{30}O_6$	412	1.4×10^{-4}
$C_{20}H_{30}O_8$	444	1.3×10^{-4}
$C_{20}H_{30}O_{10}$	476	8.4×10^{-5}
$C_{20}H_{30}O_{12}$	508	9.3×10^{-5}
$C_{20}H_{30}O_{14}$	540	4.5×10^{-5}
$C_{20}H_{30}O_{16}$	572	2.5×10^{-5}
$C_{20}H_{30}O_{18}$	604	7.7×10^{-6}
$C_{18}H_{26}O_4$ ($C_{13}H_{22}O_8$)	352	2.8×10^{-4}
$C_{19}H_{28}O_5$	382	4.0×10^{-4}
$C_{19}H_{28}O_7$	414	3.2×10^{-4}
$C_{19}H_{28}O_9$	446	3.0×10^{-4}
$C_{19}H_{28}O_{11}$	478	2.2×10^{-4}
$C_{12}H_{20}O_3$	258	4.6×10^{-4}
$C_{13}H_{22}O_4$	288	2.6×10^{-4}
$C_{13}H_{22}O_6$	320	9.1×10^{-5}

* signal(adduct) / (signal($C_2H_5NH_3^+$) + signal(($C_2H_5NH_2$)$C_2H_5NH_3^+$))
background corrected; duty-cycle corrected

Table S4: Main signals of accretion products ($C_3H_7NH_3^+$ adducts) and their intensities from the ozonolyis of α-pinene measured in the free-jet flow system at TROPOS, re-analysis of published data in ref. 4, see the spectrum Fig. S4 of this work. Signals at nominal 364, 380 and 394 Th remained unidentified.

Composition	Peak No.	Nominal adduct mass (product)$C_3H_7NH_3^+$ (Th)	Normalized signal *
$C_{20}H_{30}O_6$	7	426	3.7×10^{-4}
$C_{20}H_{30}O_8$	12	458	2.5×10^{-4}
$C_{20}H_{30}O_{10}$	17	490	1.9×10^{-4}
$C_{20}H_{30}O_{12}$	22	522	1.4×10^{-4}
$C_{20}H_{30}O_{14}$	27	554	6.2×10^{-5}
$C_{20}H_{30}O_{16}$	29	586	3.6×10^{-5}
$C_{20}H_{30}O_{18}$	30	618	1.0×10^{-5}
$C_{20}H_{34}O_4$	4	398	1.2×10^{-3}
$C_{20}H_{34}O_6$	9	430	3.1×10^{-4}
$C_{20}H_{34}O_8$	14	462	2.8×10^{-4}
$C_{20}H_{34}O_{10}$	19	494	8.8×10^{-5}
$C_{20}H_{34}O_{12}$	24	526	4.4×10^{-5}
$C_{20}H_{32}O_5$	5	412	6.6×10^{-4}
$C_{20}H_{32}O_7$	10	444	5.0×10^{-4}
$C_{20}H_{32}O_9$	15	476	3.8×10^{-4}
$C_{20}H_{32}O_{11}$	20	508	1.8×10^{-4}
$C_{20}H_{32}O_{13}$	25	540	9.3×10^{-5}
$C_{20}H_{32}O_{15}$	28	572	3.8×10^{-5}
$C_{18}H_{26}O_4$ §	1	366	1.2×10^{-3}
$C_{19}H_{28}O_5$	3	396	1.2×10^{-3}
$C_{19}H_{28}O_7$	8	428	5.2×10^{-4}
$C_{19}H_{28}O_9$	13	460	5.5×10^{-4}
$C_{19}H_{28}O_{11}$	18	492	3.3×10^{-4}
$C_{19}H_{28}O_{13}$	23	524	2.5×10^{-5}
$C_{19}H_{30}O_4$ §	2	382	2.6×10^{-3}
$C_{19}H_{30}O_6$	6	414	1.2×10^{-3}
$C_{19}H_{30}O_8$	11	446	7.9×10^{-4}
$C_{19}H_{30}O_{10}$	16	478	$< 5\times10^{-6}$
$C_{19}H_{30}O_{12}$	21	510	1.8×10^{-5}
$C_{19}H_{30}O_{14}$	26	542	$< 5\times10^{-6}$

* signal(adduct) / (signal($C_2H_5NH_3^+$) + signal(($C_2H_5NH_2$)$C_2H_5NH_3^+$))
background corrected; duty-cycle corrected

§ likely influenced by processes within the IMR

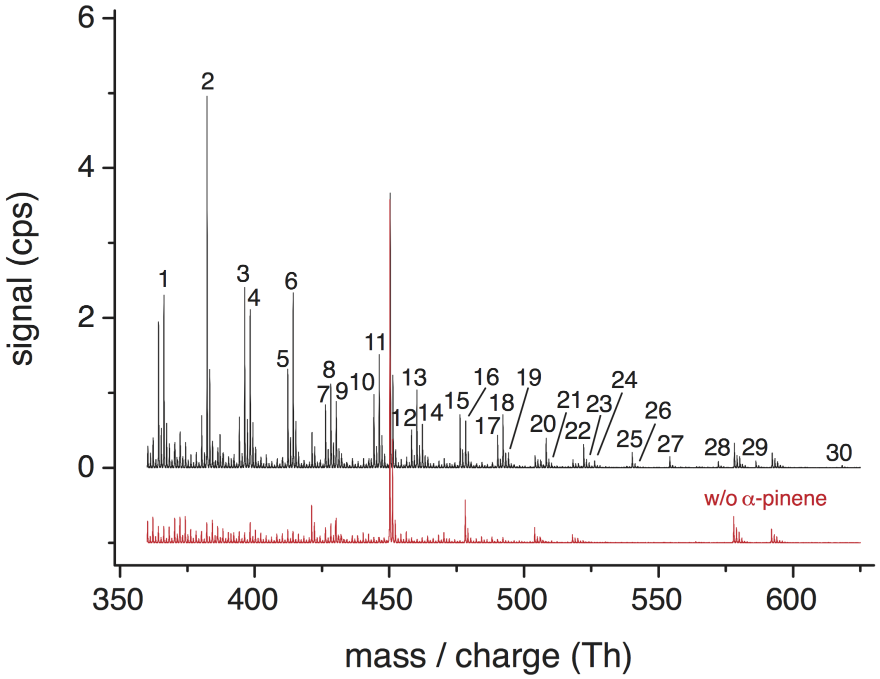

Figure S4: Spectrum of accretion products ($C_3H_7NH_3^+$ adducts) in the C_{18}-C_{20} range from the ozonolyis of α-pinene measured in the free-jet flow system at TROPOS, re-analysis of data published in ref. 4, see Figures 4 and S8 and Table S1 there. Corresponding peak composition and intensity of main signals are given in Table S4. Reactant concentrations were: [α-pinene] = 1.2 $\times 10^{12}$ and [O_3] = 6.4 $\times 10^{11}$ molecules cm^{-3}.

Table S5: Main signals of accretion products ($C_3H_7NH_3^+$ adducts) and their intensities from the ozonolyis of α-pinene in the presence of ethylene measured in the free-jet flow system at TROPOS, re-analysis of published data in ref. 4, see the spectrum Fig. S5 of this work.

Composition	Peak No.	Nominal adduct mass (product)$C_3H_7NH_3^+$ (Th)	Normalized signal *
$C_{20}H_{30}O_6$	8	426	2.0×10^{-4}
$C_{20}H_{30}O_8$	10	458	1.3×10^{-4}
$C_{20}H_{30}O_{10}$	12	490	1.1×10^{-4}
$C_{20}H_{30}O_{12}$	14	522	1.0×10^{-4}
$C_{20}H_{30}O_{14}$	15	554	4.3×10^{-5}
$C_{20}H_{30}O_{16}$	16	586	2.4×10^{-5}
$C_{20}H_{30}O_{18}$	17	618	8.7×10^{-6}
$C_{20}H_{34}O_4$	5	398	2.4×10^{-4}
$C_{18}H_{26}O_4$§	1	366	1.1×10^{-3}
$C_{19}H_{28}O_5$	4	396	7.5×10^{-4}
$C_{19}H_{28}O_7$	9	428	3.7×10^{-4}
$C_{19}H_{28}O_9$	11	460	3.7×10^{-4}
$C_{19}H_{28}O_{11}$	13	492	2.3×10^{-4}
$C_{19}H_{30}O_4$§	3	382	3.8×10^{-4}
$C_{19}H_{30}O_6$	7	414	1.9×10^{-4}
$C_{11}H_{18}O_4$	-	274	2.1×10^{-3}
$C_{12}H_{20}O_5$	-	304	8.4×10^{-4}
$C_{12}H_{20}O_7$	-	336	2.3×10^{-4}
$C_{12}H_{20}O_9$	2	368	2.5×10^{-4}
$C_{12}H_{20}O_{11}$	6	400	1.3×10^{-4}

* signal(adduct) / (signal($C_2H_5NH_3^+$) + signal(($C_2H_5NH_2$)$C_2H_5NH_3^+$))
background corrected; duty-cycle corrected

§ likely influenced by processes within the IMR

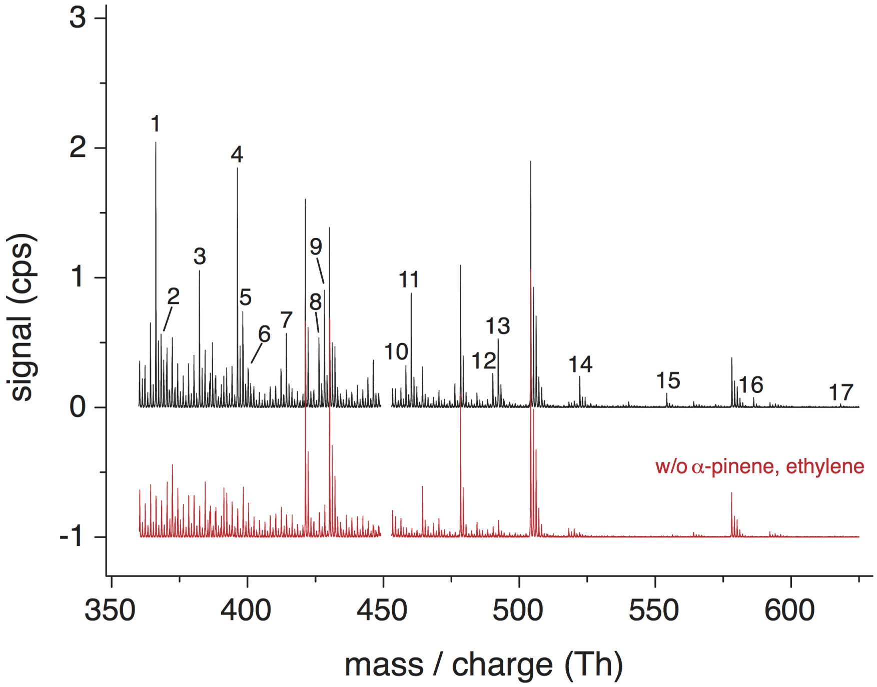

Figure S5: Spectrum of accretion products ($C_3H_7NH_3^+$ adducts) in the C_{18}-C_{20} range from the ozonolyis of α-pinene in the presence of ethylene measured in the free-jet flow system at TROPOS, re-analysis of data published in ref. 4. Corresponding peak composition and intensity of main signals are given in Table S5. Reactant concentrations were: [α-pinene] = 3.9×10^{11} , [ethylene] = 8.03×10^{12} and [O_3] = 2.0×10^{12} molecules cm^{-3}.

Table S6: Main signals of accretion products ($C_3H_7NH_3^+$ adducts) and their intensities from the ozonolyis of α-pinene in the presence of isoprene measured in the free-jet flow system at TROPOS, re-analysis of published data in ref. 4, see the spectrum Fig. S6 of this work.

Composition	Peak No.	Nominal adduct mass (product)$C_3H_7NH_3^+$ (Th)	Normalized signal *
$C_{20}H_{30}O_6$	8	426	1.7×10^{-4}
$C_{20}H_{30}O_8$	11	458	1.3×10^{-4}
$C_{20}H_{30}O_{10}$	13	490	1.3×10^{-4}
$C_{20}H_{30}O_{12}$	15	522	1.1×10^{-4}
$C_{20}H_{30}O_{14}$	16	554	4.8×10^{-5}
$C_{20}H_{30}O_{16}$	17	586	2.6×10^{-5}
$C_{20}H_{30}O_{18}$	18	618	7.6×10^{-6}
$C_{20}H_{34}O_4$	5	398	9.4×10^{-5}
$C_{18}H_{26}O_4$§	1	366	7.8×10^{-4}
$C_{19}H_{28}O_5$	4	396	6.9×10^{-4}
$C_{19}H_{28}O_7$	9	428	3.4×10^{-4}
$C_{19}H_{28}O_9$	12	460	3.8×10^{-4}
$C_{19}H_{28}O_{11}$	14	492	2.6×10^{-4}
$C_{19}H_{30}O_4$§	3	382	1.9×10^{-4}
$C_{19}H_{30}O_6$	7	414	9.3×10^{-5}
$C_{14}H_{22}O_4$	-	314	2.9×10^{-4}
$C_{15}H_{24}O_5$	-	344	4.2×10^{-4}
$C_{15}H_{24}O_7$	2	376	9.7×10^{-5}
$C_{15}H_{24}O_9$	6	408	8.9×10^{-5}
$C_{15}H_{24}O_{11}$	10	440	5.2×10^{-5}

* signal(adduct) / (signal($C_2H_5NH_3^+$) + signal(($C_2H_5NH_2$)$C_2H_5NH_3^+$))
background corrected; duty-cycle corrected

§ likely influenced by processes within the IMR

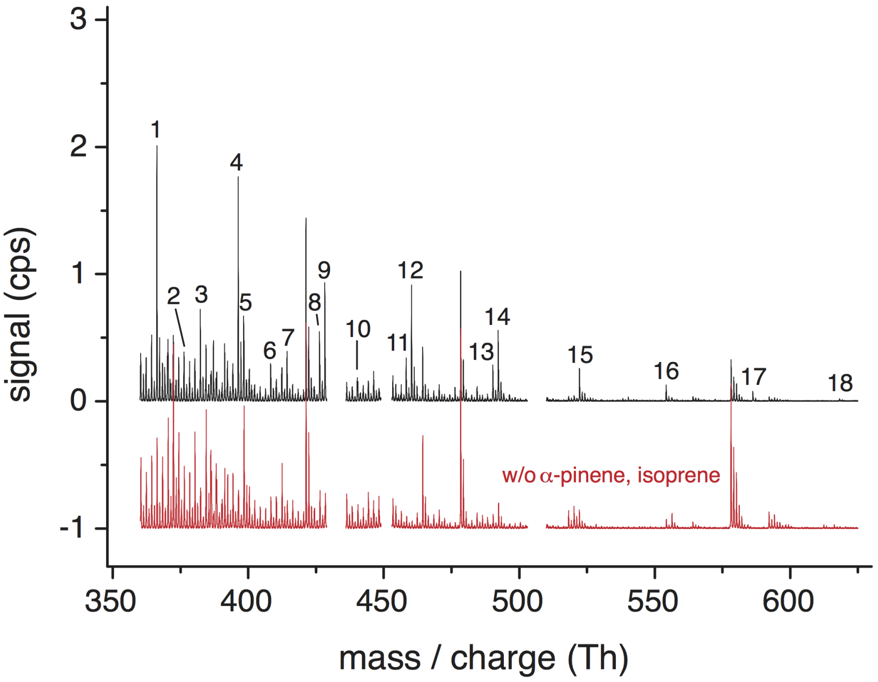

Figure S6: Spectrum of accretion products ($C_3H_7NH_3^+$ adducts) in the C_{18}-C_{20} range from the ozonolyis of α-pinene in the presence of isoprene measured in the free-jet flow system at TROPOS, re-analysis of data published in ref. 4. Corresponding peak composition and intensity of main signals are given in Table S6. Reactant concentrations were: [α-pinene] = 3.9×10^{11}, [isoprene] = 1.46×10^{12} and [O_3] = 2.0×10^{12} molecules cm^{-3}.

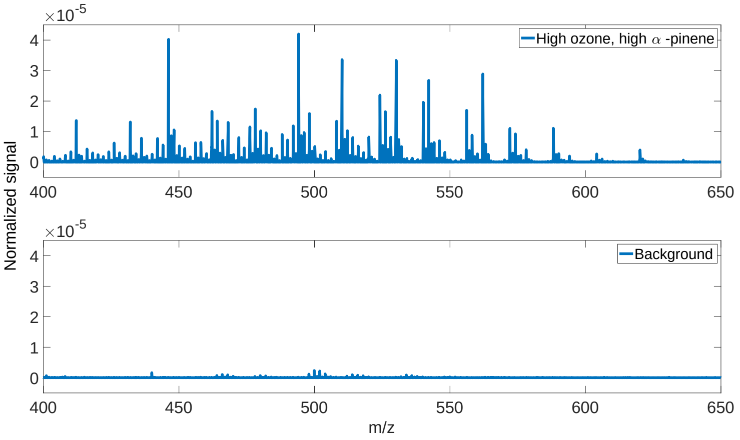

Figure S7: Spectrum of accretion products (NO_3^- adducts) in the C_{18}-C_{20} range from the ozonolyis of α-pinene the COALA chamber. Corresponding peak composition and intensity of main signals are given in Table S7. Reactant concentrations were: [α-pinene] = 5×10^{11} and [O_3] = 2.3×10^{12} molecules cm^{-3}.

Table S7: Main signals of accretion products (NO_3^- adducts) and their intensities from the ozonolyis of α-pinene measured in COALA chamber, spectra in Figs. 3a and 3e in the main text and spectrum in Fig. S7.

Composition	Nominal adduct mass (product)NO_3^- (Th)	Normalized signal *
$C_{19}H_{28}O_{11}$	494	13.7×10^{-4}
$C_{20}H_{32}O_{13}$	542	11.6×10^{-4}
$C_{20}H_{32}O_{11}$	510	11.1×10^{-4}
$C_{18}H_{28}O_{16}$	562	10.4×10^{-4}
$C_{19}H_{28}O_{8}$	446	8.4×10^{-5}
$C_{20}H_{30}O_{12}$	524	8.3×10^{-5}
$C_{18}H_{28}O_{14}$	530	8.2×10^{-5}
$C_{20}H_{30}O_{14}$	556	7.9×10^{-5}
$C_{20}H_{30}O_{16}$	588	7.7×10^{-5}
$C_{20}H_{30}O_{13}$	540	7.2×10^{-5}
$C_{20}H_{30}O_{15}$	572	5.6×10^{-5}
$C_{19}H_{28}O_{13}$	526	4.9×10^{-5}
$C_{19}H_{28}O_{12}$	510	4.7×10^{-5}
$C_{19}H_{28}O_{9}$	462	4.1×10^{-5}
$C_{20}H_{32}O_{15}$	574	4.0×10^{-5}
$C_{20}H_{30}O_{18}$	620	4.0×10^{-5}
$C_{20}H_{32}O_{12}$	526	3.9×10^{-5}
$C_{18}H_{28}O_{12}$	498	3.8×10^{-5}
$C_{20}H_{30}O_{11}$	508	3.7×10^{-5}
$C_{19}H_{28}O_{10}$	478	3.5×10^{-5}

* signal(adduct) / (signal(NO_3^-) + signal((HNO_3)NO_3-)) + signal((HNO_3)$_2$$NO_3-$))

Table S8: A listing of the ten accretion products with the highest signals in the TROPOS flow tube, in rough order of magnitude (highest first). Reactant concentrations were $[O_3] = 6.4 \times 10^{11}$ molecules cm^{-3} and $[\alpha\text{-pinene}] = (1.08 - 12.1) \times 10^{11}$ molecules cm^{-3}. Along with the detected accretion product, we also list tentative RO_2 participating in its formation. For accretion products with higher oxygen numbers, multiple combinations of reactant RO_2 are possible. Also listed is whether we assume a beta-scission has occurred in the formation, and the tentative accretion product type. Based on reanalysis of data given in Berndt et al.,[4] measured with $C_3H_7NH_3^+$ ionization.

Compound	R'O$_2$	R"O$_2$	beta-scission	Accretion product type
$C_{19}H_{28}O_5$	$C_{10}H_{15}O_4$	$C_{10}H_{15}O_4$	yes	R(O)OR
$C_{18}H_{26}O_4$	$C_{10}H_{15}O_4$	$C_{10}H_{15}O_4$	yes, both RO_2	RR
$C_{19}H_{30}O_8$	$C_{10}H_{15}O_4$	$C_{10}H_{17}O_7$	yes	R(O)OR
$C_{19}H_{28}O_9$	$C_{10}H_{15}O_4$	$C_{10}H_{15}O_8$	yes	R(O)OR
$C_{19}H_{28}O_7$	$C_{10}H_{15}O_4$	$C_{10}H_{15}O_6$	yes	R(O)OR
$C_{20}H_{32}O_7$	$C_{10}H_{15}O_{4,6}$	$C_{10}H_{17}O_{5,3}$	no	ROOR
$C_{20}H_{32}O_9$	$C_{10}H_{15}O_{4,6,8}$	$C_{10}H_{17}O_{7,5,3}$	no	ROOR
$C_{20}H_{30}O_6$	$C_{10}H_{15}O_4$	$C_{10}H_{15}O_4$	no	ROOR
$C_{19}H_{28}O_{11}$	$C_{10}H_{15}O_4$	$C_{10}H_{15}O_{10}$	yes	R(O)OR
$C_{20}H_{34}O_8$	$C_{10}H_{17}O_{3,5,7}$	$C_{10}H_{17}O_{7,5,3}$	no	ROOR

4 Quantum chemical calculations

4.1 Methods

4.1.1 Conformational Sampling of Isolated alkoxy and alkyl radicals

Systematic conformer searches for both $C_{10}O_3$ alkoxy ("RO") and C_9O_2 alkyl radical ("R") "monomer" systems were performed using the Spartan 18 program.[11] In these searches, every non-terminal bond was rotated a certain number of degrees (120 for sp^3 hybridized atoms and 180 for sp^2 hybridized atoms). A molecular mechanics force field (MMFF) optimization was performed to find distinct minimum energy structures on the PES. We found 11 conformers for the alkoxy (RO) and 8 conformers for the alkyl (R) radical monomers. The number of unpaired electrons was set to one, and the total charge were set to zero. Note that unlike earlier versions of Spartan, where peroxy radicals were sometimes treated as anions when default force-field settings were used, Spartan 18 does not require manually setting the type of the radical oxygen atom (e.g. to generic divalent oxygen).

4.1.2 Systematic sampling of the ^{3}(RO$\cdots$OR) and ^{3}(R$\cdots$OR) clusters

Systematic conformational sampling of ^{3}(RO$\cdots$OR) and ^{3}(R$\cdots$OR) clusters was done using the building up approach described by Kubečka et al..[12] The purpose of this sampling was to find representative local minima structure on the potential energy surface (PES). The details of the sampling are similar to our previous work on smaller ^{3}(RO$\cdots$OR) clusters.[12,13] In brief, the monomer conformers from the previous steps were taken and optimized at the ωb97X-D/6-311++G** level of theory. Afterward, the Artificial Bee Colony (ABC) algorithm[14,15] was used to generate thousands of cluster structures for the both ^{3}(RO$\cdots$OR) and ^{3}(R$\cdots$OR) clusters. The ABC algorithm performs rigid-body molecular dynamics, which requires partial charges and Lennard Jones parameters for each of the atoms in the cluster. Partial charges were obtained from natural bonding orbital (NBO) population calculations at the ωb97X-D/6-311++G** level, while Lennard-Jones[16] parameters were taken from the CHARMM force field database.[17] Three thousand cluster conformers were initially generated by the ABC algorithm for each combination of monomer conformers. For 11 alkoxy ("RO") conformers, there are in total 66 possible distinct pairs, leading to 198 000 initial conformers for the ^{3}(RO$\cdots$OR) cluster. Similarly, the 11 RO and 8 R conformers (88 distinct pairs) led to a total of 264 000 initial conformers for the ^{3}(R$\cdots$OR) cluster.

Next, GFN-xTB semiempirical optimization was performed for all the conformers generated from ABC using the XTB program.[18] Especially in the ^{3}(R$\cdots$OR) cluster, various unwanted reactions (e.g. recombination) happened during the XTB optimization due a combination of strained input geometries, and possibly low reaction barriers (at least at this level of theory). While some of these reactions may be real, they are undesirable artifacts from the point of view of the conformational sampling of ^{3}(R$\cdots$OR) clusters. We first tested different optimization criteria available in the XTB program to maximize the number of intact conformers for our higher level (DFT) calculations. However, this was unsuccessful. We therefore used a systematic filtering approach to remove the unwanted conformers which appeared as minima in the XTB PES. We used not only the radius of gyration and

electronic energies as collective coordinates, but specific bond lengths (for example H...C, O...C, and H...O bonds corresponding to the unwanted reactions) using the Goodvibes program implemented in a Python module.[19] After removing all redundant conformers, as well as conformers with bond lengths characteristic of products of unwanted reactions, we selected all conformers within 10 kcal/mol of the lowest-energy conformer for lower-level DFT optimizations at the ωb97X-D/6-31+G* level of theory[20,21] using Gaussian 16, Revision B.01.[22] Filtering was then again performed based on electronic energy and dipole moment. Conformers within 5 kcal/mol of the lowest-energy conformed were then optimized (and vibrational frequencies were calculated) at the ωb97X-D/6-31++G** level of theory.

4.1.3 Sampling of the ROR ester accretion product

About 2703 conformers of the ROR ester accretion product, generated by MMFF conformational sampling in Spartan 18 as described above, were optimized using two different semiempirical methods. First, the GFN-xTB method was applied on both the triplet and singlet surfaces using the XTB program.[18] Redundant and high-energy conformers were then filtered out using the approach described above. (Various unwanted conformers, corresponding to artifact reactions, were found on the triplet surface, and manually discarded). Second, the same set of conformations was sampled using the ODM3 method,[23] as implemented in the MNDO2020 semiempirical code,[24] on both singlet and triplet surfaces. On the triplet surface, approximately one third of the optimizations failed to converge due to failure of the SCF procedure. Despite this, we were able to obtain a large number (on the order of a thousand) of optimized low-energy configurations on both surfaces. The 20 best (lowest-energy) conformers each were selected from the singlet and triplet surfaces from both semiempirical methods for DFT optimization at the ωb97X-D/6-31++G** level. Recombination energetics were then calculated for both singlet and triplet conformers.

4.1.4 ISC rate Calculation

The global minimum-energy conformer, and one representative local minimum conformer with a clearly different bonding pattern, were selected from the ωb97X-D/6-31++G** optimized ^{3}(RO$\cdots$OR) and ^{3}(R$\cdots$OR) cluster structures for the inter-system crossing (ISC) rate constant calculation. We used the XMC-QDPT2 multireference method since we are working with open-shell systems.[25,26] The details of the calculations are described in our previous work.[13,27] Briefly, the ISC rate constant (k_{ISC}) was calculated using the following formula:[28]

$$k_{\mathrm{ISC}}(T_i \rightarrow S_j) = 1.6 \times 10^9 \times \langle \phi(T_i)|\hat{H}_{\mathrm{SO}}|\phi(S_j)\rangle^2 \times F_{ij}, \quad (1)$$

where $\langle \phi(T_i)|\hat{H}_{\mathrm{SO}}|\phi(S_j)\rangle^2$ are the spin orbit coupling matrix elements (SOCME) in cm^{-1}, and F_{ij} is the Franck-Condon factor for the transition. Both singlet and triplet energies were calculated using the XMC-QDPT2/6-311++G(d,p) level of theory in Firefly, version 8.2.0, 2016.[29] For the ^{3}(RO$\cdots$OR) cluster, we used the (6,4) active space (six electrons in four molecular orbitals). For the ^{3}(RO...R) cluster, test calculations indicated that a larger active space was needed, and we used ten electrons in ten molecular orbitals (10,10). The justification for choosing the (6,4) active space for the ^{3}(RO$\cdots$OR) cluster was that the main orbitals contributing to the included states (4 singlets and 4 triplets) were HOMO, HOMO-1, HOMO-2 and LUMO (Fig. S8).

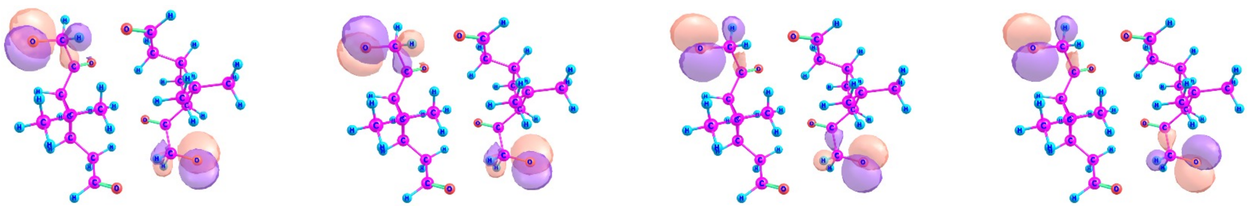

HOMO HOMO-1 HOMO-2 LUMO

Figure S8: State averaged CASSF (6,4)/6-311++G(d,p) molecular orbitals for the global minima conformers of the ^{3}(RO$\cdots$OR) cluster.

Similarly, the (10,10) active space was chosen for the 3(RO...R) cluster as the main contributing orbitals (to the 4 singlet and 4 triplet states) were HOMO, HOMO-1, HOMO-

2, HOMO-3, HOMO-4, LUMO, LUMO+1, LUMO+2, LUMO+3 and LUMO+4 (Fig. S9).

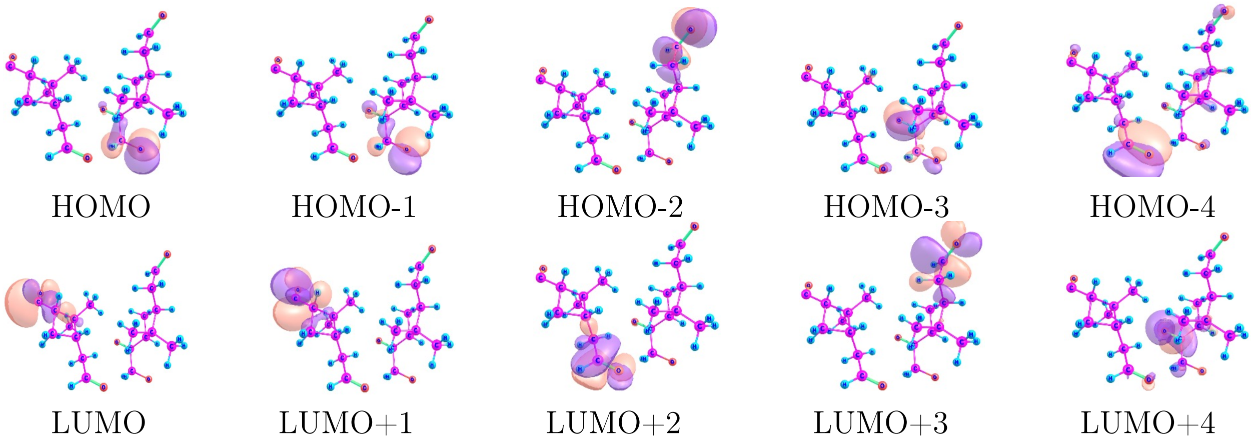

Figure S9: State averaged CASSF (10,10)/6-311++G(d,p) molecular orbitals for the global minima conformers of the ^{3}(RO. . . R) cluster.

The matrix element of spin-orbital coupling interaction (SOCME) between triplet states T1-T4 and singlet states S1-S4 were computed at the CASSCF level of theory, using the XMC-QDPT2/6-311++G(d,p) energies.[30] We used GAMESS-US for this calculation.[31]

4.1.5 Alkoxy Bond Scission Rate Calculation

In addition to these calculations, the rate of the RO_2-Kb derived alkoxy bond scission reaction was determined computationally to further explain our experimental results of the C_{19} ester being the main product. Conformer sampling was performed according to the scheme described in Section 4.1.1. Conformers of the transition state were sampled with the scissioning C-C bond frozen after an initial saddle point structure was found, after which the same optimization and filtering workflow was applied as for the alkoxy radicals. All alkoxy radical conformers of suitably low energy were optimized at the ωB97X-D/aug-cc-pVTZ level of theory, after which a transition state structure was searched for by taking the lowest-energy conformer and streching the scissioning OC-C=O bond length to 2.0 Å, perfoming a low-level constrained optimization (B3LYP/6-31+G*) and performing another round of conformational sampling for the formed structure. The energetically lowest structures were again optimized at the ωB97X-D/aug-cc-pVTZ level of theory, after which the

reaction rate was calculated using the Eyring equation (2), in this case neglecting quantum tunneling as it is highly unlikely to impact a reaction dependent of heavy atom motion at 300 K, and as calculation of a tunneling coefficient would have required optimization of the formed $C_9H_{13}O_2$ radical as well.

$$k_{\text{scission}} = \frac{k_B T}{h} \frac{Q_{\text{TS}}}{Q_{\text{RO}}} \exp\left\{ \frac{E_{\text{RO}} - E_{\text{TS}}}{k_B T} \right\} \tag{2}$$

where RO and TS refer to the alkoxy radical and bond scission transition state, respectively. k_B is naturally the Boltzmann constant, h is Planck's constant, T is the temperature, and Q_i are the canonical partition functions of the noted chemical structure, both calculated using Frequency analysis in Gaussian 16 at the ωB97X-D/aug-cc-pVTZ level.

Hindered rotor scans were performed on the final ωB97X-D/aug-cc-pVTZ geometries, successfully for the alkoxy radical and unsuccessfully for the transition state. This resulting correction factor for the reactant partition function Q_R was 1.50, which at the very least suggests that bond rotations do not have an order of magnitude impact on the reaction rate.

4.2 Results and discussion

The stability of both $^3(\text{RO}\cdots\text{OR})$ and $^3(\text{R}\cdots\text{OR})$ clusters are shown in the Table S9. The binding energies of the two clusters are roughly comparable to each other, and also to the larger of the $^3(\text{RO}\cdots\text{OR})$ clusters investigated in our previous study.[13] The weaker electronic binding energy of the $^3(\text{R}\cdots\text{OR})$ cluster is probably related to the smaller number of C-H...O interactions, as evident from Fig. S10. However, this is partially compensated by the more favorable entropy of the looser $^3(\text{R}\cdots\text{OR})$ structure, resulting in almost identical Gibbs free energies of dissociation. Using the detailed-balance approach in our previous study, these free energies would imply cluster dissociation rates on the order of 10^{11} s^{-1}. However, as discussed extensively in the same study, this is almost certainly an overestimate of the true dissociation rate.

The model does not account for the impact of the dissociation energy of the radical pair, which likely slows the dissociation down by several orders of magnitude. The dissociation energy of the triplet state (RO$\cdots$OR') is 14 kcal/mol (Table S9). A dissociation energy this high means the dissociation rate is going to be substantially lower than 10^{11} s^{-1}. Using a novel first-principles model[32] on calculating the dissociation rate, we can estimate it to be in the range of 10^4 s^{-1} range, several orders of magnitude below 10^{11} s^{-1}.

To verify that the relatively low binding energies (and thus high detailed-balance evaporation rates) are not caused by conformer issues (i.e. the neglect of low-lying conformers), or by errors in the commonly employed rigid rotor-harmonic oscillator approximation, we have carried out additional test calculations. First, we recomputed all thermal contributions to the free energies of all species using the quasi-harmonic approximation.[33] This was especially important for the ^{3}(RO$\cdots$OR) system, as the lowest free-energy conformer had multiple vibrational modes with wavenumbers on the order of 10 cm^{-1}. Next, we recomputed the free energies for the ^{3}(RO$\cdots$OR) => RO + RO and ^{3}(R$\cdots$OR) => RO + R reactions using all cluster conformers within 2 kcal/mol in free energy, and the approach of Partanen et al.[34] to account for multiple conformers. The resulting free energies were -2.2 kcal/mol for the ^{3}(RO$\cdots$OR) => RO + RO reaction, and -4.7 kcal/mol for the ^{3}(R$\cdots$OR) => RO + R reaction. As these free energies would still imply detailed-balance evaporation rates in excess of 10^{11} s^{-1} for both systems, we can conclude that neither conformer effects nor errors in the harmonic oscillator entropies are behind the qualitative failure of the detailed-balance approach.

Table S9: Relative Electronic Energies and Gibbs Free Energies (at 298 K and 1 atm) for the dissociation reactions of the studied clusters: ^{3}(RO...OR) → RO + RO and ^{3}(R$\cdots$OR) → R + RO, computed at the ωb97X-D/6-31++G** level of Theory.

Cluster	ΔE (kcal/mol)	ΔG (kcal/mol)
^{3}(RO$\cdots$OR)	+ 13.682	-3.166
^{3}(R$\cdots$OR)	+ 10.620	-2.992

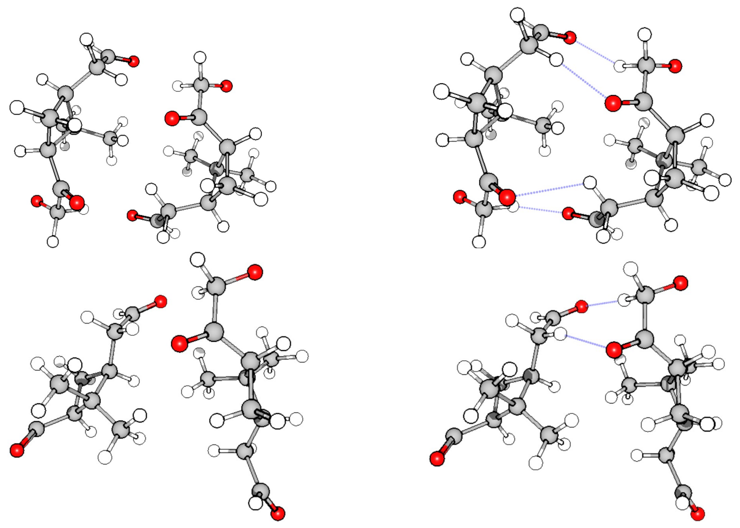

Figure S10: Optimized structures of the global minimum-energy conformers of the ^{3}(RO$\cdots$OR) (top) and ^{3}(R$\cdots$OR) (bottom) clusters. (The right-hand-side structures denotes all the closest H...O interactions. Note that these are not H-bonds according to most conventional definitions, as the H atoms are bonded to C atoms.) The distance between the radical centers is 6.7 Å in the ^{3}(RO$\cdots$OR) cluster and 8.9 Å in the ^{3}(R$\cdots$OR) cluster.

4.2.1 ISC rate calculation

ISC rate coefficients (from the triplet to the singlet surface) for the studied two conformers of the ^{3}(RO$\cdots$OR) and ^{3}(R$\cdots$OR) clusters are given in Table S10.

Table S10: ISC rate constant for ^{3}(RO$\cdots$OR) and ^{3}(R$\cdots$OR) clusters. Computed using XMC-QDPT2^a/6-311++G(d,p) energies, with matrix elements of the Spin-Orbit Coupling Interaction (SOCME) between T_1-T_4 and S_1-S_4 states computed at the CASSCF^a/6-311++G(d,p) level.

Clusters	Conformers	$\sum K_{\mathrm{ISC}}$ (s^{-1})
^{3}(RO$\cdots$OR)	b	1.29×10^7
	c	1.77×10^8
^{3}(R$\cdots$OR)	b	1.00×10^8
	c	7.00×10^{11}

^{a} (6,4) and (10,10) active spaces were used for the ^{3}(RO$\cdots$OR) and ^{3}(R$\cdots$OR) clusters, respectively. ^{b} global minima conformer ^{c} a representative local minima conformer.

For both of ^{3}(RO$\cdots$OR) and ^{3}(R$\cdots$OR) clusters, we computed the ISC rate both for the global minima conformers, as well as another representative low-energy conformer with different bonding pattern, in order to see how the rates changes between conformers.

Our results confirmed that the ISC rate in both clusters are reasonably fast, similar to our previous study on smaller ^{3}(RO$\cdots$OR) clusters, though with a large variation between conformers.[13] Notably, the rates predicted for the ^{3}(R$\cdots$OR) cluster are even faster than for the ^{3}(RO$\cdots$OR) cluster, and the rate for the second ^{3}(R$\cdots$OR) conformer exceeds even the upper limit dissociation rate estimated by the detailed balance approach. Thus, provided that the ^{3}(R$\cdots$OR) cluster forms in the first place (e.g. due to rapid alkoxy scission in the ^{3}(RO$\cdots$OR) cluster), ISC to the singlet state is very likely a competitive pathway.

4.2.2 Stability of the ROR ester accretion product

The recombination energetics of ROR ester accretion product (with respect to the free RO and R radicals) were calculated on both singlet and triplet surfaces (Table S11). Unsurprisingly, recombination on the singlet surface is extremely exergonic. However, also recombination on the triplet surface was found to be exergonic, due to the presence of carbonyl groups and thus low-lying triplet states in the ester product. Thus, direct recombination of ^{3}(R$\cdots$OR) to ^{3}(ROR) is thermodynamically possible – albeit likely associated with an energy barrier making it uncompetitive with either ISC or dissociation (Fig. S11).

Table S11: Relative Electronic Energies and Gibbs Free Energies (at 298 K and 1 atm) for the formation of the ROR accretion products from the free RO and R radicals, calculated at ωB97X-D/6-31++G** level of theory.

ROR accretion product	ΔE in kcal/mol	ΔG in kcal/mol
Singlet	– 104.56	– 82.547
Triplet	– 30.459	– 9.517

4.2.3 Alkoxy Bond Scission Rate Calculation

Using Eq. (2), we calculated the rate for the beta scission of the RO derived from RO_2-Kb to be 2×10^9 s^{-1}. The optimized structure for the scissioning RO, and the transition state towards the scissioned products, are shown in Fig. S13.

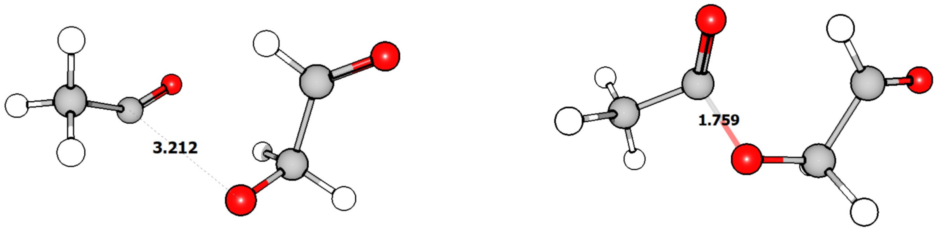

Radical complex Transition state

Figure S11: Optimized structures (at the ωB97X-D/aug-cc-pVTZ level) of a representative transition state connecting a $CH_3{-}C\cdot(O)\ldots\cdot O{-}CH_2{-}CH(O)$ alkyl-alkoxy radical complex to the corresponding ester, on the triplet surface. The electronic energy (free energy at 298 K and 1 atm) difference between the two is 13.6 (11.5) kcal/mol. Note that a full conformer sampling could not be performed for this reaction, as e.g. transition state conformer guesses with the aldehyde oxygen in a "cis" position led to other transition states interconverting different covalently bound diradical species. As these transition states did not connect (through IRC paths) to the correct reactant complex, their existence should not change the qualitative conclusion that direct recombination of alkyl and alkoxy radicals on the triplet surface is likely associated with a substantial barrier.

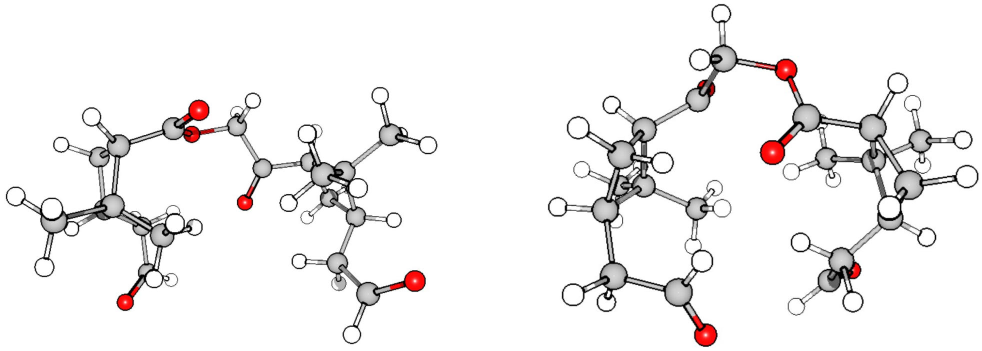

Singlet Triplet

Figure S12: Optimized structures of ROR ester accretion product on both singlet (left) and triplet (right) surfaces.

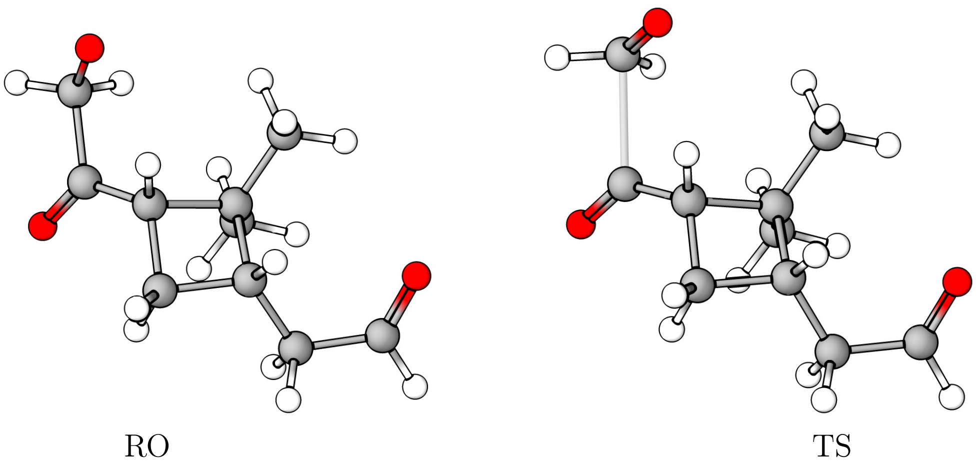

Figure S13: ωB97X-D/aug-cc-pVTZ optimized structures for the scissioning RO derived from RO_2-Kb and the transition state (TS) towards the scissioned products, CH_2O and $C_9H_{13}O_2$

5 Interpretation of the D shift spectra: labile H, max number of abstracted D from CD3 AP.

5.1 Exchange of labile deuteriums to hydrogen with H_2O

Even though we did not humidify the COALA chamber for the majority of the experiments, enough water vapour was present in the system to exchange all the labile deuteriums to hydrogens. We verified this by actively humidifying the chamber during the oxidation of deuterated α-pinene. The deuteration shifted certain HOM compounds by a number of mass units, often fewer than three, as compared to the non-deuterated case. This indicates that the HOM had lost deuterium during oxidation, most probably through intramolecular hydrogen abstraction in autoxidation. Through this, the deuterium atoms became bound to hydroperoxide groups, and thus labile. No change in the spectrum was observed during humidification, meaning that all of the labile deuteriums were already exchanged in the non-humidified chamber (Fig. S14).

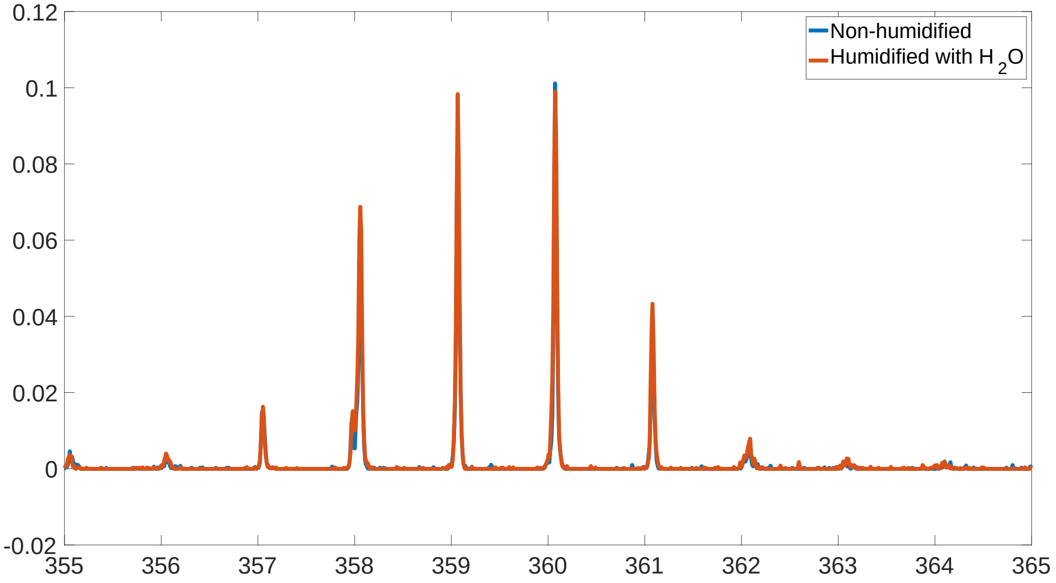

Figure S14: Example HOM spectrum in the reaction of the deuterated α-pinene with ozone. The shown signal consists mainly of HOM compounds $C_{10}H_{14}O_{10}$, $C_{10}H_{15}O_{10}$ and $C_{10}H_{16}O_{10}$, with varying degrees of deuteration. At least some have lost a deuterium atom in autoxidation (main text), meaning that it has become attached to a hydroperoxide group (in this case -OOD). Upon humidification with H_2O, virtually no change in the spectrum is observed. This means that all of the labile deuteriums (-OOD) have already been exchanged to hydrogen in the dry case. All peaks are observed clustered with the nitrate ion.

5.2 Maximum number of deuterium lost in the autoxidation of d3-α-pinene

Any deuterium atoms abstracted during autoxidation are exchanged with H_2O for ordinary hydrogen. This means that the loss of a deuterium atom from an observed compound means that that specific deuterium has been abstracted during autoxidation. The most highly oxygenated RO_2 radicals produced in autoxidation have the formula $C_{10}H_{15}O_{10}$: these form $C_{20}H_{30}O_{18}$ accretion products in their self reactions.[4] As compared to ordinary α-pinene, $C_{20}H_{30}O_{18}$ accretion products formed from the oxidation of d3-α-pinene shift by four or five mass units (Fig. S15). This means that they have lost either one or two deuteriums as compared to the total of six originally present in the two precursor α-pinene molecules. The loss of one deuterium is explained by one of the reacting RO_2 radicals losing one deuterium, while the loss of two could be a result of both losing one, or one RO_2 losing two, while the other has lost none. However, if the latter was the case, we would also expect RO_2s that have lost two deuterium atoms to react with others that have lost one or two as well, resulting in $C_{20}H_{30}O_{18}$ accretion products that have lost more than two deuterium atoms. As these are not observed, we can deduce that up to one of the three deuterium atoms in the d3-α-pinene can be lost in autoxidation. Thus, for the deuterated version of $C_{19}H_{28}O_{11}$ that has lost a total of four, it is very likely that one has been lost in autoxidation, and the remaining three have been lost together with the carbon atom they were bound to.

6 Non-ideal behaviour of certain accretion products in dilution experiments

The experiments at TROPOS were conducted using a dilution system for the inlet flow of the CI-APi-TOF. A dilution with nitrogen by up to a factor of seven was performed before the inlet of the instrument. If no unwanted processes occur inside the inlet, and the mea-

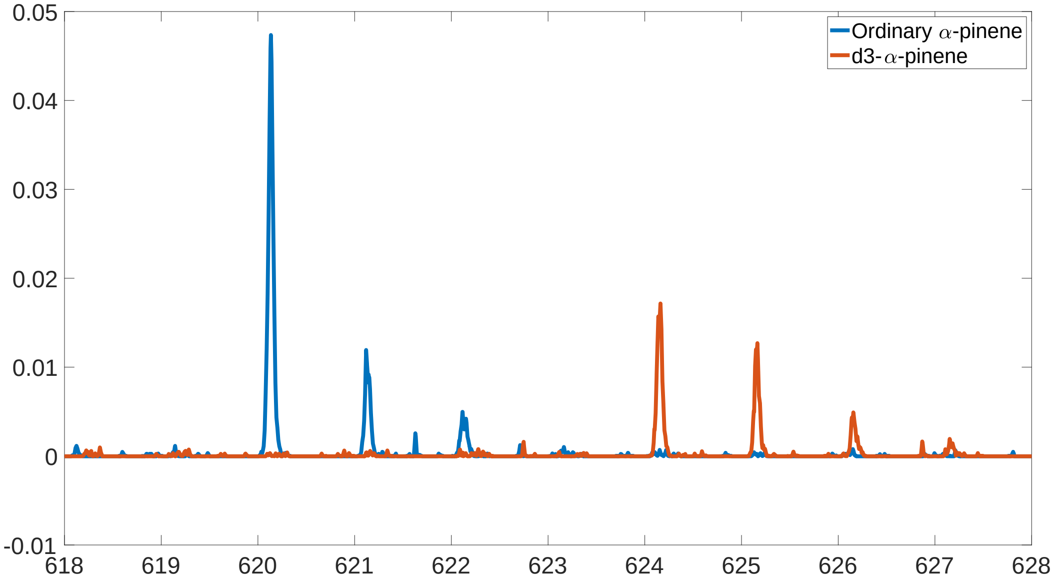

Figure S15: HOM spectra in the reaction of the deuterated and non-deuterated α-pinene with ozone. The shown signal consists of HOM accretion product $C_{20}H_{30}O_{18}$, formed in the reaction of two $C_{10}H_{15}O_{10}$, or its deuterated forms. The shift of at least four Da when switching from normal to deuterated α-pinene means that at least four deuterium atoms remain in the product, and a maximum of two have been abstracted during autoxidation. This implies a maximum of one D-abstraction per reacting $C_{10}H_{15}O_{10}$. All peaks are observed clustered with the nitrate ion.

sured signal comes from products formed already before the inlet, the signals observed with the instrument should decrease by a factor of seven. The majority of the measured accretion products, RO_2 radicals and closed shell monomeric species followed this ideal dilution behaviour. However, some compounds, especially the accretion products $C_{18}H_{26}O_4$ (partly overlapping with another ion, Fig. S16), $C_{19}H_{30}O_4$, and to a lesser extent, $C_{19}H_{28}O_5$, and the RO_2 radical $C_{10}H_{15}O_4$, did not follow this behaviour. Specifically, the accretion products decrease by a factor larger than seven, up to 22, and the RO_2 radical decreases by a smaller factor. This may be explained in terms of ion-induced clustering: for these relatively little oxidized RO_2, clustering with the ethylaminium ion in the inlet may increase their reactivity towards each other. This would lead to disproportionately fast RO_2-RO_2 reactions within the inlet, resulting in the depletion of the reactant RO_2 and excess production of the product accretion products. The extent of this effect would be proportional to the product of the concentrations of the reactant RO_2: hence, the effect diminishes with increasing dilution. To verify whether this is the case, we performed further computational modelling in the next section.

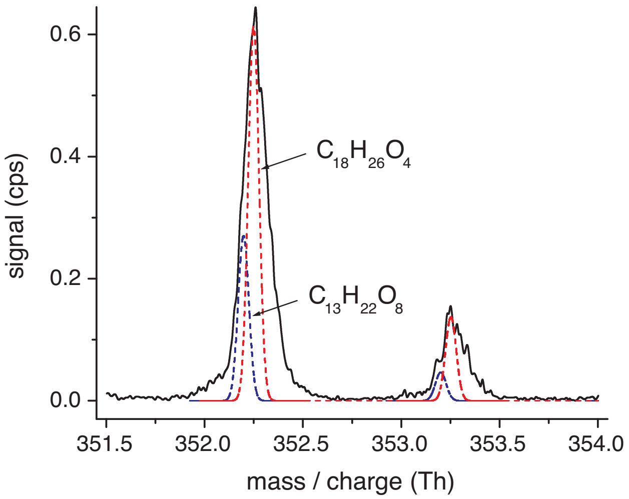

Figure S16: Product spectrum of the α-pinene ozonolysis in the range 351.5 - 354.0 Th obtained using ethylaminium ionization. The product signal appears as an adduct with $C_2H_5NH_3^+$ and is shifted by 46.09 Th. The spectrum represents the measured raw signal recorded from 10 min data accumulation. The red trace is the signal of $(C_{18}H_{26}O_4)C_2H_5NH_3^+$ from peak fitting and the blue trace the signal of $(C_{13}H_{22}O_8)C_2H_5NH_3^+$.

6.1 Molecular Dynamics Simulations of Interactions Between α-Pinene Derived Peroxy Radicals and the Ethyl-ammonium Cation $CH_3CH_2NH_3^+$

We have previously modelled the association lifetimes of the pre-reactive complex formed by two peroxy radicals by simulating collisions between them using empirical force fields (for details of the force field models, simulation procedures, and results, we refer the reader to our previous publications[35,36]). In Daub et al. we calculated the lifetime for the association of two RO_2-Ka to be 247 ps.[36] Based on this, and our observed correlation between the complex lifetimes and known reaction rates for a variety of other peroxy radicals, we predict a reaction rate for tetroxide formation in the reaction of two RO_2-Ka of $k_{\text{pred}} = 428 \times 10^{-13}$ cm^3 molec.$^{-1}$ s^{-1} (Table S12). The corresponding numbers for two RO_2-Kb are 145 ps and 251×10^{-13} cm^3 molec.$^{-1}$ s^{-1} . For a cross reaction of RO_2-Ka with RO_2-Kb, we calculated the association time to be 221 ps.

Table S12: Association time of complexes between RO_2 species and predicted reaction rates based on ref. 36.

Reacting RO_2's	Association time (ns)	Reaction rate predicted from association time[36] (cm^3 molec.$^{-1}$ s^{-1})
RO_2-Ka +RO_2-Ka	247	428×10^{-13}
RO_2-Kb +RO_2-Kb	145	251×10^{-13}
RO_2-Ka +RO_2-Kb	221	

To study the influence of the ethyl-ammonium cation, we first optimized the structure of the complex between a single RO_2-Kb and $CH_3CH_2NH_3^+$ by running molecular dynamics simulations at low temperature (5 K). The structure with the lowest binding energy is shown in Figure S17. This structure shows the formation of very strong hydrogen bonds between the ammonium group and the oxygens in the peroxy radical, leading to a binding energy of -28 kcal/mol. Then, we simulated collisions between the RO_2-Kb + ethylammonium ionic complex and another RO_2-Kb. In each of the 100 collisions simulated, we observed the formation of an associated complex of all three molecules which remained held together for

the entire 2 ns of the simulation. Based on this, and assuming that any energetic barriers for tetroxide formation are not significantly altered by the presence of the cation, we can predict that the overall reactivity of the cation-bound complex should be essentially collision-limited. This is supported by the structure of the lowest energy tri-molecular complex, also shown in Figure S17. When the second α-pinene peroxy is included, the interaction energy gets lowered by an additional 20 kcal/mol, for a total association energy of -48 kcal/mol.

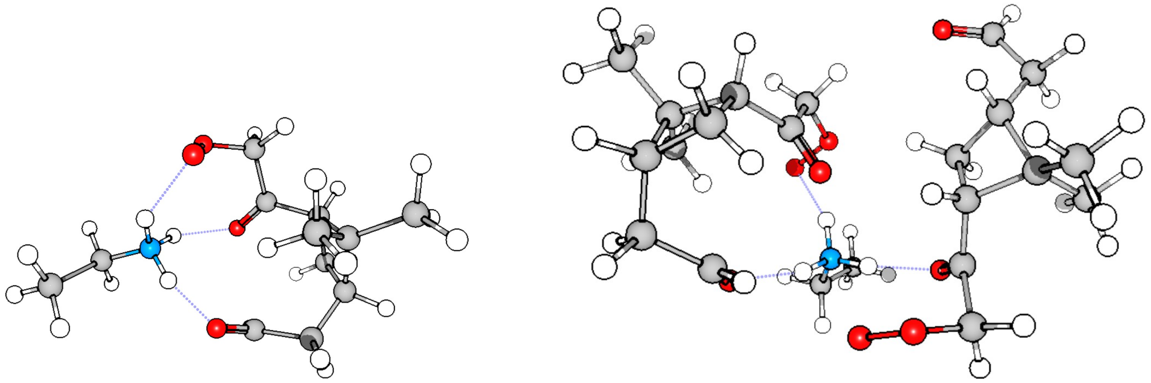

Figure S17: Left) Minimum energy configuration of RO_2-Kb + $CH_3CH_2NH_3^+$. Right) Minimum energy configuration of 2 RO_2-Kb + $CH_3CH_2NH_3^+$. Configurations are from LAMMPS simulations run at 5 K.

In summary, the presence of the ethylammonium ion stabilizes the clusters, likely increasing reaction rates. Further, it seems that without the ion, the clusters of two RO_2-Kb are more weakly bound as compared to clusters of two RO_2-Ka. Clusters between RO_2-Ka and RO_2-Kb lie between the two. As a result, the reaction rate of two RO_2-Kb is farther away from collision limited, and there is more room for its ion enhancement. This fits qualitatively with our results, where $C_{18}H_{26}O_4$ were the most affected by ion mediated processes, while $C_{19}H_{28}O_5$ was only slightly affected, and $C_{20}H_{30}O_6$ not at all. The first is expected to form from the reaction between two RO_2-Kb, second in e.g. reaction of RO_2-Ka with RO_2-Kb, and the last e.g. in the reaction of two RO_2-Ka.

7 Scissions of other RO

We have extensively studied the potential beta scission of the RO derived from RO_2-Kb (main text Fig. 1). In addition to RO_2-Kb, α-pinene ozonolysis is also know to product at least four other RO_2 radicals with the formula $C_{10}H_{15}O_4$, some of which can undergo autoxidation to lead to an even larger variety of RO_2.[37,38] In addition, OH-oxidation of α-pinene produces RO_2-radicals with the formula $C_{10}H_{17}O_3$ (Fig. S1), again some of which can undergo autoxidation.[3,39] When these types of RO_2 react together, forming the RO$\cdots$OR' complex, they too may undergo scission reactions while still in the complex. We studied the scissions available to the RO derived from the four other $C_{10}H_{15}O_4$ RO_2 radicals from α-pinene ozonolysis in main text Fig. 1 (RO_2-I, RO_2-Ka, RO_2-Kc and RO_2-Kd), and the three $C_{10}H_{17}O_3$ RO_2 radicals shown in Fig. S1 (known as APINAO2, APINBO2 and APINCO2).

Table S13: Scission rates for the RO scission reactions shown in Fig. S18. The rates are calculated using the SAR by Vereecken and Peeters.[40]

Isomer	SAR rate (s^{-1})	Closed-shell product	Radical Product
RO_2-I	3×10^8	C_3H_6O	$C_7H_9O_2$
RO_2-Ka	1×10^8	$C_9H_{14}O_2$	CHO
RO_2-Kc,d (R1)	9×10^9	$C_8H_{12}O_2$	C_2H_3O
RO_2-Kc,d (R2)	1×10^{13}		$C_{10}H_{15}O_3$
APINAO2	1×10^{11}		$C_{10}H_{17}O_2$
APINBO2	3×10^{11}		$C_{10}H_{17}O_2$
APINCO2	4×10^8	C_3H_6O	$C_7H_{11}O$

Based on the SAR,[40] we found fast scission reactions for all of the studied RO radicals. In some cases, such as for RO_2-I, the scission would lead to fragmentation of the molecule: in this case it would leave a $C_7H_9O_2$ alkyl radical product. Sometimes the radical product would be small, such as for RO_2-Ka where a CHO alkyl radical is formed. In other cases, the scission would break a ring structure, without fragmenting the molecule in parts. An example of this would be the extremely fast scission available to RO_2-Kc and RO_2-Kd, leaving a $C_{10}H_{15}O_3$ alkyl radical. If such a radical would recombine with a non-scissioned ten-carbon RO from α-pinene ozonolysis, the resulting product would be an ether (ROR) bonded C_{20}

accretion product. Further, if two RO_2-Kc react together, it is conceivable that they would, after scission on both sides, form a C_{20} accretion product with a carbon bound backbone.

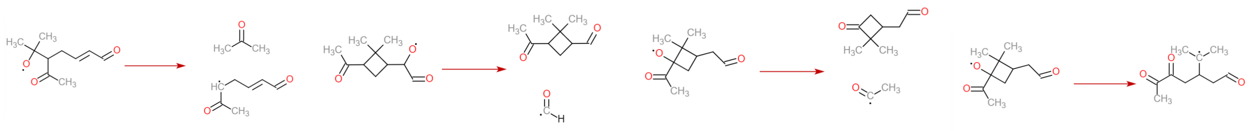

RO_2-I　　RO_2-Ka　　RO_2-K(c,d), 1　　RO_2-K(c,d), 2

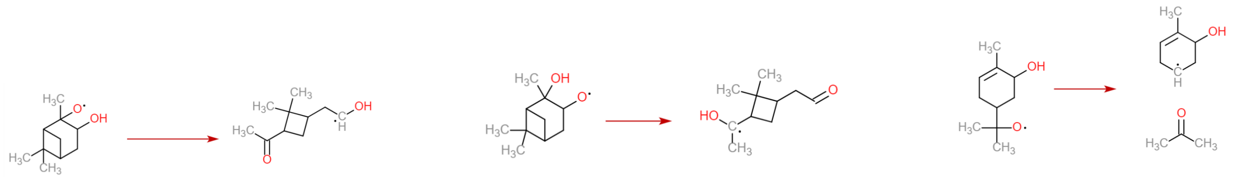

APINAO2　　APINBO2　　APINCO2

Figure S18: The scission products for the RO radicals derived from the first-generation RO_2 radicals formed in the ozone (top row) or OH radical (bottom row) oxidation of α-pinene. RO_2-Kc and RO_2-Kd are stereoisomers, and have the same potential scission points. These isomers have two competing alkoxy bond scission reactions, of which the much faster one breaks the 4-carbon ring, thus having no closed-shell products: both reactions are shown.

References

(1) Vereecken, L.; Francisco, J. Theoretical studies of atmospheric reaction mechanisms in the troposphere. *Chemical Society Reviews* **2012**, *41*, 6259–6293.

(2) Xu, L.; Møller, K. H.; Crounse, J. D.; Otkjær, R. V.; Kjaergaard, H. G.; Wennberg, P. O. Unimolecular Reactions of Peroxy Radicals Formed in the Oxidation of α-Pinene and β-Pinene by Hydroxyl Radicals. *The Journal of Physical Chemistry A* **2019**, *123*, 1661–1674.

(3) Rolletter, M.; Kaminski, M.; Acir, I.-H.; Bohn, B.; Dorn, H.-P.; Li, X.; Lutz, A.; Nehr, S.; Rohrer, F.; Tillmann, R.; Wegener, R.; Hofzumahaus, A.; Kiendler-Scharr, A.; Wahner, A.; Fuchs, H. Investigation of the α-pinene photooxidation by OH in the atmospheric simulation chamber SAPHIR. *Atmospheric Chemistry and Physics* **2019**, *19*, 11635–11649.

(4) Berndt, T.; Mentler, B.; Scholz, W.; Fischer, L.; Herrmann, H.; Kulmala, M.; Hansel, A. Accretion Product Formation from Ozonolysis and OH Radical Reaction of α-Pinene: Mechanistic Insight and the Influence of Isoprene and Ethylene. *Environmental Science & Technology* **2018**, *52*, 11069–11077.

(5) Berndt, T.; Kaethner, R.; Voigtländer, J.; Stratmann, F.; Pfeifle, M.; Reichle, P.; Sipilä, M.; Kulmala, M.; Olzmann, M. Kinetics of the unimolecular reaction of CH_2OO and the bimolecular reactions with the water monomer, acetaldehyde and acetone under atmospheric conditions. *Phys. Chem. Chem. Phys.* **2015**, *17*, 19862–19873.

(6) Berndt, T.; Hyttinen, N.; Herrmann, H.; Hansel, A. First oxidation products from the reaction of hydroxyl radicals with isoprene for pristine environmental conditions. *Communications Chemistry* **2019**, *2*, 21.

(7) Lindinger, W.; Hansel, A.; Jordan, A. On-line monitoring of volatile organic compounds at pptv levels by means of proton-transfer-reaction mass spectrometry (PTR-MS) medical applications, food control and environmental research. *International Journal of Mass Spectrometry and Ion Processes* **1998**, *173*, 191–241.

(8) Eisele, F. L.; Tanner, D. J. Ion-assisted tropospheric OH measurements. *Journal of Geophysical Research: Atmospheres* **1991**, *96*, 9295–9308.

(9) Riva, M.; Heikkinen, L.; Bell, D.; Peräkylä, O.; Zha, Q.; Schallhart, S.; Rissanen, M.; Imre, D.; Petäjä, T.; Thornton, J.; Zelenyuk, A.; Ehn, M. Chemical transformations in monoterpene-derived organic aerosol enhanced by inorganic composition. *npj Climate and Atmospheric Science* **2019**, *2*, 2.

(10) Peräkylä, O.; Riva, M.; Heikkinen, L.; Quéléver, L.; Roldin, P.; Ehn, M. Experimental investigation into the volatilities of highly oxygenated organic molecules (HOMs). *Atmospheric Chemistry and Physics* **2020**, *20*, 649–669.

(11) Wavefunction Inc., Spartan 18. 2018; Irvine, CA.

(12) Kubečka, J.; Besel, V.; Kurtén, T.; Myllys, N.; Vehkamäki, H. Configurational Sampling of Noncovalent (Atmospheric) Molecular Clusters: Sulfuric Acid and Guanidine. *The Journal of Physical Chemistry A* **2019**, *123*, 6022–6033.

(13) Hasan, G.; Salo, V.-T.; Valiev, R. R.; Kubečka, J.; Kurtén, T. Comparing Reaction Routes for ^{3}(RO···OR′) Intermediates Formed in Peroxy Radical Self- and Cross-Reactions. *The Journal of Physical Chemistry A* **2020**, *124*, 8305–8320.

(14) Zhang, J.; Dolg, M. ABCluster: the artificial bee colony algorithm for cluster global optimization. *Physical Chemistry Chemical Physics* **2015**, *17*, 24173–24181.

(15) Zhang, J.; Dolg, M. Global optimization of clusters of rigid molecules using the artificial bee colony algorithm. *Physical Chemistry Chemical Physics* **2016**, *18*, 3003–3010.

(16) Glendening, E.; Reed, A.; Carpenter, J.; Weinhold, F. NBO, version 3.1. 2003; Gaussian Inc. Wallingford CT.

(17) Vanommeslaeghe, K.; Hatcher, E.; Acharya, C.; Kundu, S.; Zhong, S.; Shim, J.; Darian, E.; Guvench, O.; Lopes, P.; Vorobyov, I.; Mackerell, A. D. CHARMM general force field: A force field for drug-like molecules compatible with the CHARMM all-atom additive biological force fields. *Journal of Computational Chemistry* **2009**,

(18) Grimme, S.; Bannwarth, C.; Shushkov, P. A Robust and Accurate Tight-Binding Quantum Chemical Method for Structures, Vibrational Frequencies, and Noncovalent Interactions of Large Molecular Systems Parametrized for All spd-Block Elements (Z = 1 – 86). *Journal of Chemical Theory and Computation* **2017**, *13*, 1989–2009.

(19) Funes-Ardois, I.; Paton, R. Goodvibes: Goodvibes v1.0.1. 2016; `http://dx.doi.org/10.5281/zenodo.60811`.

(20) Lee, C.; Yang, W.; Parr, R. G. Development of the Colle-Salvetti correlation-energy formula into a functional of the electron density. *Physical Review B* **1988**, *37*, 785–789.

(21) Hehre, W. J.; Ditchfield, R.; Pople, J. A. Self—Consistent Molecular Orbital Methods. XII. Further Extensions of Gaussian—Type Basis Sets for Use in Molecular Orbital Studies of Organic Molecules. *The Journal of Chemical Physics* **1972**, *56*, 2257–2261.

(22) Frisch, M. J. et al. Gaussian 16 Revision B.01. 2016; Gaussian Inc. Wallingford CT.

(23) Dral, P. O.; Wu, X.; Thiel, W. Semiempirical Quantum-Chemical Methods with Orthogonalization and Dispersion Corrections. *Journal of Chemical Theory and Computation* **2019**, *15*, 1743–1760.

(24) Thiel, W. MNDO2020 package. 2020; Max-Planck-Institut für Kohlenforschung: Mülheim an der Ruhr, Germany.

(25) Vereecken, L.; Glowacki, D. R.; Pilling, M. J. Theoretical Chemical Kinetics in Tropospheric Chemistry: Methodologies and Applications. *Chemical Reviews* **2015**, *115*, 4063–4114.

(26) Valiev, R. R.; Fliegl, H.; Sundholm, D. Bicycloaromaticity and Baird-type bicycloaromaticity of dithienothiophene-bridged [34]octaphyrins. *Physical Chemistry Chemical Physics* **2018**, *20*, 17705–17713.

(27) Valiev, R. R.; Hasan, G.; Salo, V.-T.; Kubečka, J.; Kurten, T. Intersystem Crossings Drive Atmospheric Gas-Phase Dimer Formation. *The Journal of Physical Chemistry A* **2019**, *123*, 6596–6604.

(28) Gadirov, R.; Valiev, R.; Samsonova, L.; Degtyarenko, K.; Izmailova, N.; Odod, A.; Krasnikova, S.; Yakushchenko, I.; Kopylova, T. Thermally activated delayed fluorescence in dibenzothiophene sulfone derivatives: Theory and experiment. *Chemical Physics Letters* **2019**, *717*, 53–58.

(29) Granovsky, A. Firefly, version 8.2.0. 2016; `http://classic.chem.msu.su/gran/gamess/index.html`.

(30) Valiev, R. R.; Cherepanov, V. N.; Baryshnikov, G. V.; Sundholm, D. First-principles method for calculating the rate constants of internal-conversion and intersystem-crossing transitions. *Physical Chemistry Chemical Physics* **2018**, *20*, 6121–6133.

(31) Schmidt, M. W.; Baldridge, K. K.; Boatz, J. A.; Elbert, S. T.; Gordon, M. S.; Jensen, J. H.; Koseki, S.; Matsunaga, N.; Nguyen, K. A.; Su, S.; Windus, T. L.; Dupuis, M.; Montgomery, J. A. General atomic and molecular electronic structure system. *Journal of Computational Chemistry* **1993**, *14*, 1347–1363.

(32) Franzon, L. First-principles numerical model for irreversible dissociation of alkoxy radical dimers. Atoms, Molecules and Clusters in Motion conference. 2022.

(33) Grimme, S. Supramolecular Binding Thermodynamics by Dispersion-Corrected Density Functional Theory. *Chemistry - A European Journal* **2012**, *18*, 9955–9964.

(34) Partanen, L.; Vehkamäki, H.; Hansen, K.; Elm, J.; Henschel, H.; Kurtén, T.; Halonen, R.; Zapadinsky, E. Effect of Conformers on Free Energies of Atmospheric Complexes. *The Journal of Physical Chemistry A* **2016**, *120*, 8613–8624.

(35) Daub, C. D.; Zakai, I.; Valiev, R.; Salo, V.-T.; Gerber, R. B.; Kurtén, T. Energy transfer, pre-reactive complex formation and recombination reactions during the collision of peroxy radicals. *Phys. Chem. Chem. Phys.* **2022**, *24*, 10033–10043.

(36) Daub, C. D.; Valiev, R.; Salo, V.-T.; Zakai, I.; Gerber, R. B.; Kurtén, T. Computed Pre-reactive Complex Association Lifetimes Explain Trends in Experimental Reaction Rates for Peroxy Radical Recombinations. *ACS Earth and Space Chemistry* **2022**, *6*, 2446–2452.

(37) Kurtén, T.; Rissanen, M. P.; Mackeprang, K.; Thornton, J. A.; Hyttinen, N.; Jørgensen, S.; Ehn, M.; Kjaergaard, H. G. Computational Study of Hydrogen Shifts and Ring-Opening Mechanisms in α-Pinene Ozonolysis Products. *The Journal of Physical Chemistry A* **2015**, *119*, 11366–11375.

(38) Iyer, S.; Rissanen, M. P.; Valiev, R.; Barua, S.; Krechmer, J. E.; Thornton, J.; Ehn, M.; Kurtén, T. Molecular mechanism for rapid autoxidation in α-pinene ozonolysis. *Nature Communications* **2021**, *12*, 878.

(39) Berndt, T.; Richters, S.; Jokinen, T.; Hyttinen, N.; Kurtén, T.; Otkjær, R. V.; Kjaergaard, H. G.; Stratmann, F.; Herrmann, H.; Sipilä, M.; Kulmala, M.; Ehn, M. Hydroxyl radical-induced formation of highly oxidized organic compounds. *Nature Communications* **2016**, *7*, 13677.

(40) Vereecken, L.; Peeters, J. Decomposition of substituted alkoxy radicals—part I: a generalized structure–activity relationship for reaction barrier heights. *Phys. Chem. Chem. Phys.* **2009**, *11*, 9062–9074.
